# MicroRNA signatures and Foxp3^+^ cell count correlate with relapse occurrence in follicular lymphoma

**DOI:** 10.18632/oncotarget.24987

**Published:** 2018-04-13

**Authors:** Giorgio Malpeli, Stefano Barbi, Corinna Greco, Simonetta Zupo, Anna Bertolaso, Maria Teresa Scupoli, Mauro Krampera, Paul Takam Kamga, Carlo Maria Croce, Aldo Scarpa, Alberto Zamò

**Affiliations:** ^1^ Department of Surgical Sciences, Dentistry, Gynecology and Pediatrics, Section of Surgery, University of Verona, Verona, Italy; ^2^ Department of Diagnostics and Public Health, University of Verona, Verona, Italy; ^3^ Department of Medicine, Section of Hematology, Stem Cell Research Laboratory, University of Verona, Italy; ^4^ Laboratory of Molecular Diagnostics, IRCCS-AOU San Martino-IST, Istituto Nazionale per la Ricerca sul Cancro, Genoa, Italy; ^5^ Department of Medicine, Section of Hematology, University of Verona, Verona, Italy; ^6^ Department of Molecular Virology, Immunology and Medical Genetics, Comprehensive Cancer Center, The Ohio State University, Columbus, OH, USA; ^7^ Applied Research on Cancer-Network, ARC-NET, University of Verona, Verona, Italy; ^8^ Department of Oncology, University of Torino, Torino, Italy

**Keywords:** follicular lymphoma, relapse, microRNAs, Foxp3, heterogeneity

## Abstract

First line drug treatment of follicular lymphoma (FL) patients is followed by a highly variable disease-free time before relapse in about one third of patients. No molecular marker is able to predict efficiently the risk of relapse. We investigated the expression profile of microRNAs (miRNAs) by microarrays and of the tumor microenvironment by immunohistochemistry in 26 FLs and 12 reactive lymph nodes (rLN) as reference. Twenty-nine miRNAs were differentially expressed in FLs compared to rLNs and some of them discriminated grade 1 from 3a FLs. Both FLs and rLNs displayed molecular heterogeneity. FLs grouped into two clusters mostly driven by the tumor T-cell content. Among 21 drug-treated FL patients with an average follow-up of 13.5 years, eight cases relapsed. Twenty-six miRNAs discriminated between relapsed and non-relapsed FLs. Ten miRNAs also correlated with Foxp3^+^ cells number. Notably, Foxp3^+^ cells were significantly less in relapsed patients and lower Foxp3^+^ cell number associated with shorter time-to-relapse. Foxp3^+^ cells did not co-expressed follicular helper T-cell markers and were therefore classified as regulatory T cells rather than follicular regulatory T-cells. These findings introduce new knowledge about the relationship between miRNA alterations and infiltrating immune cells and show that Foxp3^+^ cells might be predictive of disease relapse.

## INTRODUCTION

Follicular lymphoma (FL) is a low-grade B cell lymphoma usually characterized by long life expectancy and heterogeneous outcome. About one third of FL patients alternate periods of disease remission with recurrent relapses. Some FLs undergo histologic transformation to a high-grade neoplasm, a transition associated with a more aggressive clinical course and poor survival [1].

The current evaluation of the risk of relapse/resistance to multiagent chemotherapy of FL is based on clinical parameters summarized in the Follicular Lymphoma International Prognostic Index (FLIPI) while no consistent biological marker has been identified that predicts the survival or the risk of relapse after drug treatments [2]. The majority of candidate biomarkers studied in FL patients focused on morphologic features, cytogenetic abnormalities, molecular aberrations, somatic mutations and altered protein expression [3–7]. Although the new discovered somatic mutations are promising markers of prognosis in FL, to date no single hallmark or single driver mutation can be considered predictive of relapse.

Gene expression studies have revealed that both survival and progression-free disease are significantly associated with non-neoplastic immune cell gene signatures [8, 9]. According to gene expression signatures, FLs split into two groups, i.e. T-cell-rich and dendritic cell-/macrophage-rich [10–14]. In FL, T-cells number, functionality and distribution may impact patient prognosis. PD1^+^ cells associated with shorter time to relapse of FLs [13]. High number of Foxp3^+^ cells predicted improved survival of FL patients [10]. However, the association of Foxp3^+^ cells with FL outcome was questioned in other studies [2, 13].

MiRNAs play critical regulatory roles in the immune system and are necessary for proper lineage decision [15]. MiRNA biogenesis is under tight temporal and spatial control and dysregulation is associated with many human diseases [16, 17]. A direct role of miRNAs in lymphomagenesis was demonstrated by knock-out miRNA mouse models with a direct impact on B-cell lymphoma/leukemia development [18]. In particular, *miR-155*, *miR-150*, *miR-21*, cluster *miR-17-92* and *miR-34a* have pivotal role in the control of master regulator transcription factors essential in the B-cell biology [19]. *MiR-16* expression discriminated FLs with and without *BCL2* translocation [20]. MiRNA signatures in FLs were obtained from both the comparison with reactive lymph nodes (rLN) or from cells isolated from FL and rLNs [21–24]. Despite the potential relevance of miRNAs in FL biology, the association between the expression level of deregulated miRNAs and clinical parameters of FL patients has not been conclusively demonstrated yet.

The limited success of approaches applied so far for the identification of prognostic markers for FL patients has prompted us to consider the possible role of miRNA signatures. Since several miRNAs pointed to a role of immune cells, we have investigated the tumor microenvironment by means of immunohistochemistry. We have then investigated the possible association of markers typical of different immune cells with miRNA profiles and the clinical outcome of FL patients.

## RESULTS

### Increasing alterations of miRNAs expression from low to high grade FL

**Figure 1:**
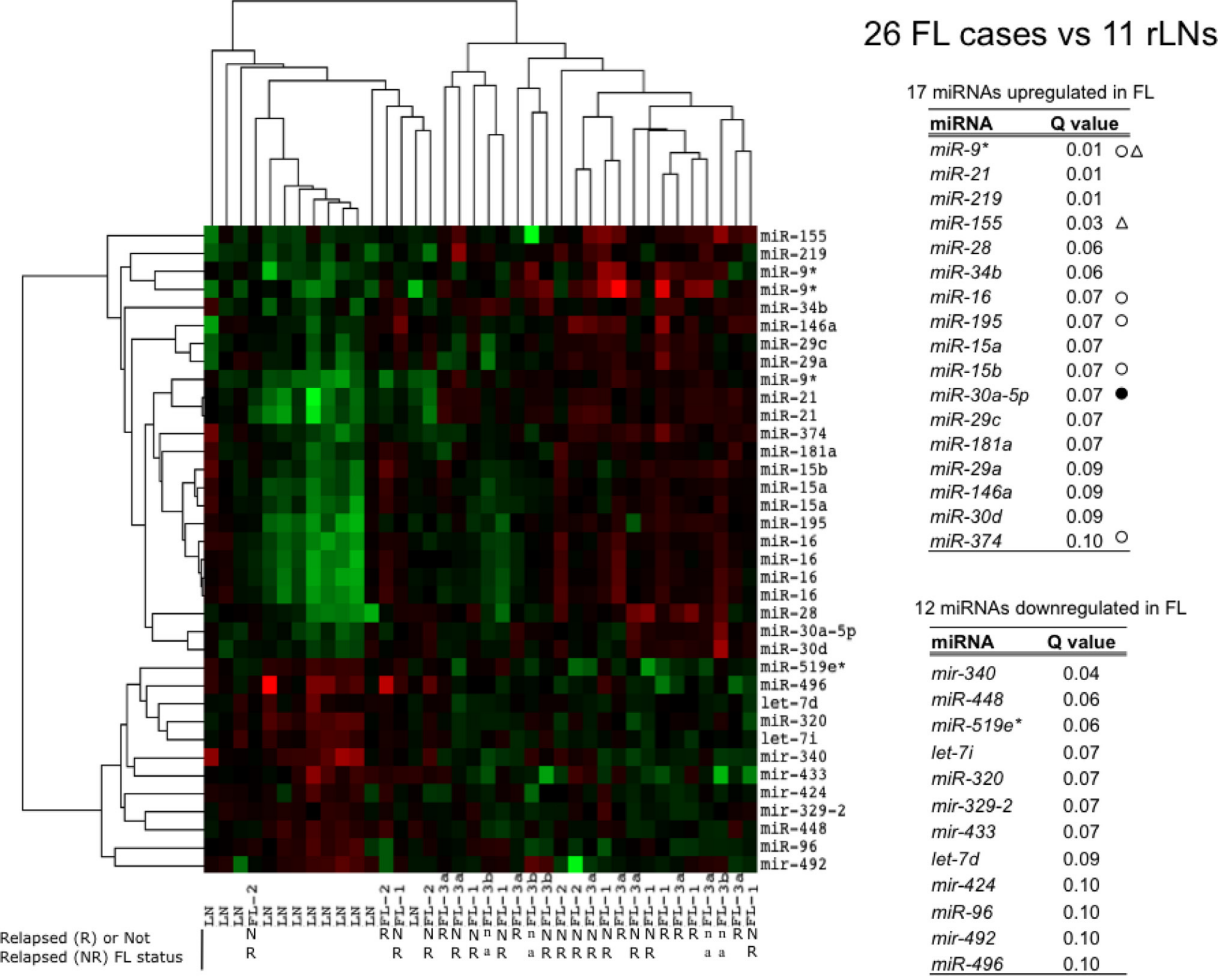
Profile of miRNAs differentially expressed between FLs and rLNs The heat map describes the expression of 36 spots, corresponding to 29 single miRNAs, differentially expressed among 38 samples: 26 follicular lymphoma (FLs) and 12 reactive lymph nodes (rLNs) (FDR 10%). FL-1, FL-2, FL-3a, FL-3b are FL of grade 1, 2, 3a and 3b, respectively. Red, higher expression (log_2_, +4); green, lower expression (log_2_, -4). The tables report the list of 17 miRNAs upregulated in FLs and of 12 miRNAs downregulated in FL in comparison to rLNs and the corresponding *Q* values. White circles: upregulated in Rohele *et al*. [23]. Black circles: downregulated in Rohele *et al.* [23]. White triangles: upregulated in isolated FL cells in Wang *et al.* [24]. R, relapsed; NR, not relapsed; n.a. not available.

We performed miRNAs expression analysis by microarrays in 26 FLs and 12 reactive lymph nodes (rLN) as reference. In Figure 1, the heat map summarizes the differences in miRNA expression between FLs and rLNs at 10% FDR (false discovery rate), fold change >1.5. FLs clustered separately from rLNs except three cases. Both FLs and rLNs appeared heterogeneous and split apart into two groups. Seventeen miRNAs resulted upregulated and 12 downregulated in FL in comparison to rLNs (Figure 1). *MiR-16* family four miRNAs, *miR-15a*, *miR-15b*, *miR-16* and *miR-195* were correlated to each other and upregulated in most of FLs.

**Figure 2:**
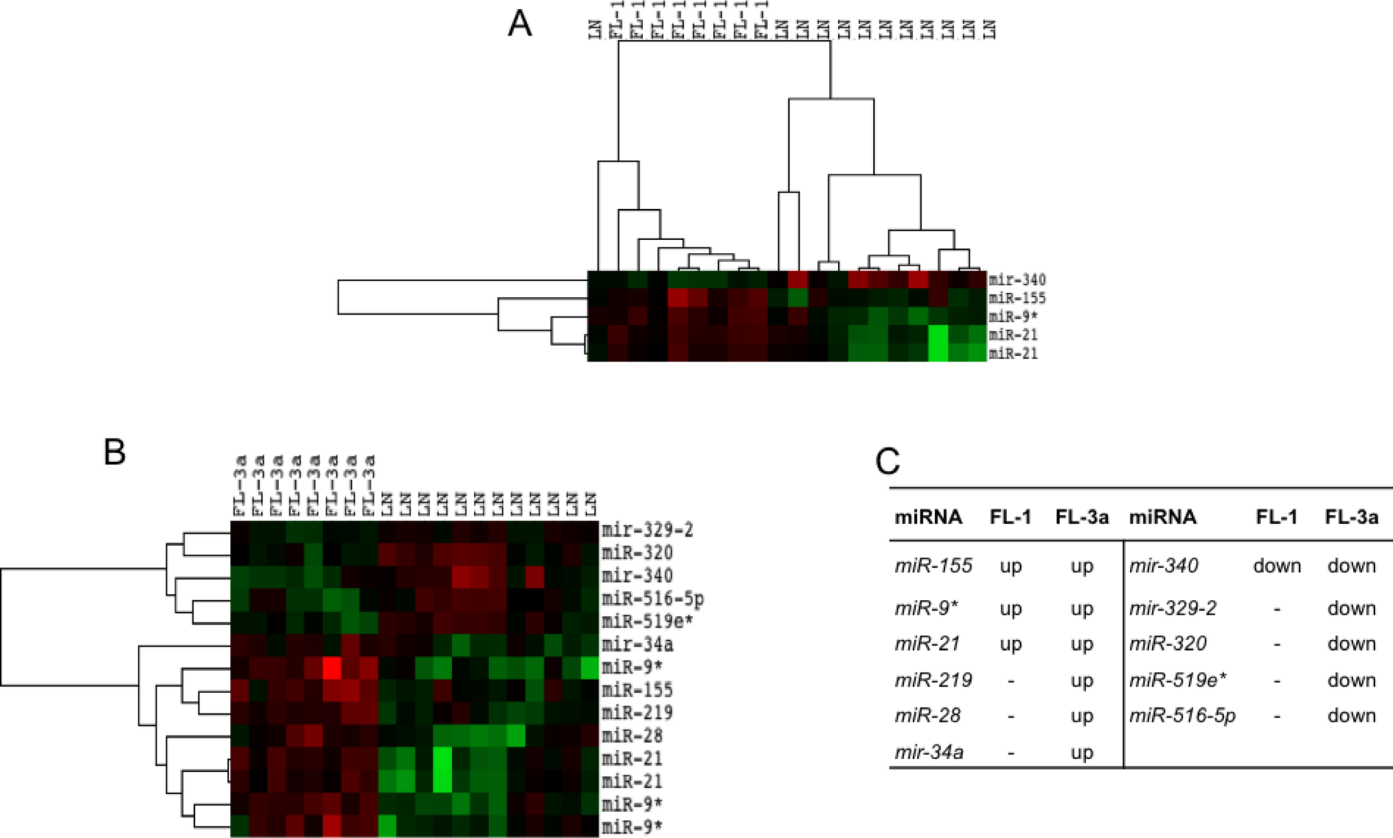
Profile of miRNAs differentially expressed between FL grade 1 or 3a and rLNs FL-1 and FL-3a are follicular lymphoma (FL) of grade 1 and 3a, respectively. Red, higher expression (log_2_, +4); green, lower expression (log_2_, –4). (**A**) The heat maps describe the expression of 14 spots, corresponding to 11 single miRNAs, differentially expressed in 20 samples: eight FLs grade 1 and 12 reactive lymph nodes (rLNs) (FDR 10%). (**B**) The heat maps describe the expression of 5 spots, corresponding to 4 single miRNAs, differentially expressed among 20 samples: eight FLs grade 3a and 12 rLNs (FDR 10%). (**C**) The table reports the list of 11 miRNAs upregulated (up) or downregulated (down) in FL grade 1 and 3a in comparison to rLNs.

FLs distributed regardless of grade as compared to rLNs. However, when FLs of grade 1, 2 and 3a were separately compared with rLNs, FL grade 1 showed the upregulation of *miR-21*, miR-9* and *miR-155* and the downregulation of *miR-340*; FL grade 3a showed the upregulation of five miRNAs and the downregulation of five miRNAs (Figure 2A and 2B). In FL grade 3, the increase of *miR-219*, *miR-28* and *miR-34a* and the decrease of *miR-329*, *miR-320*, miR-519e* and *miR-516-5p* expression added to those altered in FL grade 1 (Figure 2C). FL grade 2 showed no significant differences in comparison to rLNs as well as the direct comparison of FL of different grade.

Expression of several miRNAs was validated by quantitative RT-PCR in rLN, FL1, FL3a and all FLs (Supplementary Figure 1). *MiR-28* (*P* = 0.05), *miR-9* (*P* = 0.002), miR-9* (*P* = 0.0001), *miR-21* (*P* = 0.0007) and *miR-195* (*P* = 0.05) were confirmed to be upregulated in FLs as compared to rLN. *Mir-219* was not differentially expressed in FLs and rLN (*P* > 0.1). Downregulation of *miR-320* (*P* = 0.002), *miR-340* (*P* = 0.001) and *let-7i*(*P* = 0.05) in FLs was confirmed.

### Ratio between expression of 5p and 3p forms of miR-9 and miR-21 is different in FLs compared to rLNs

**Figure 3:**
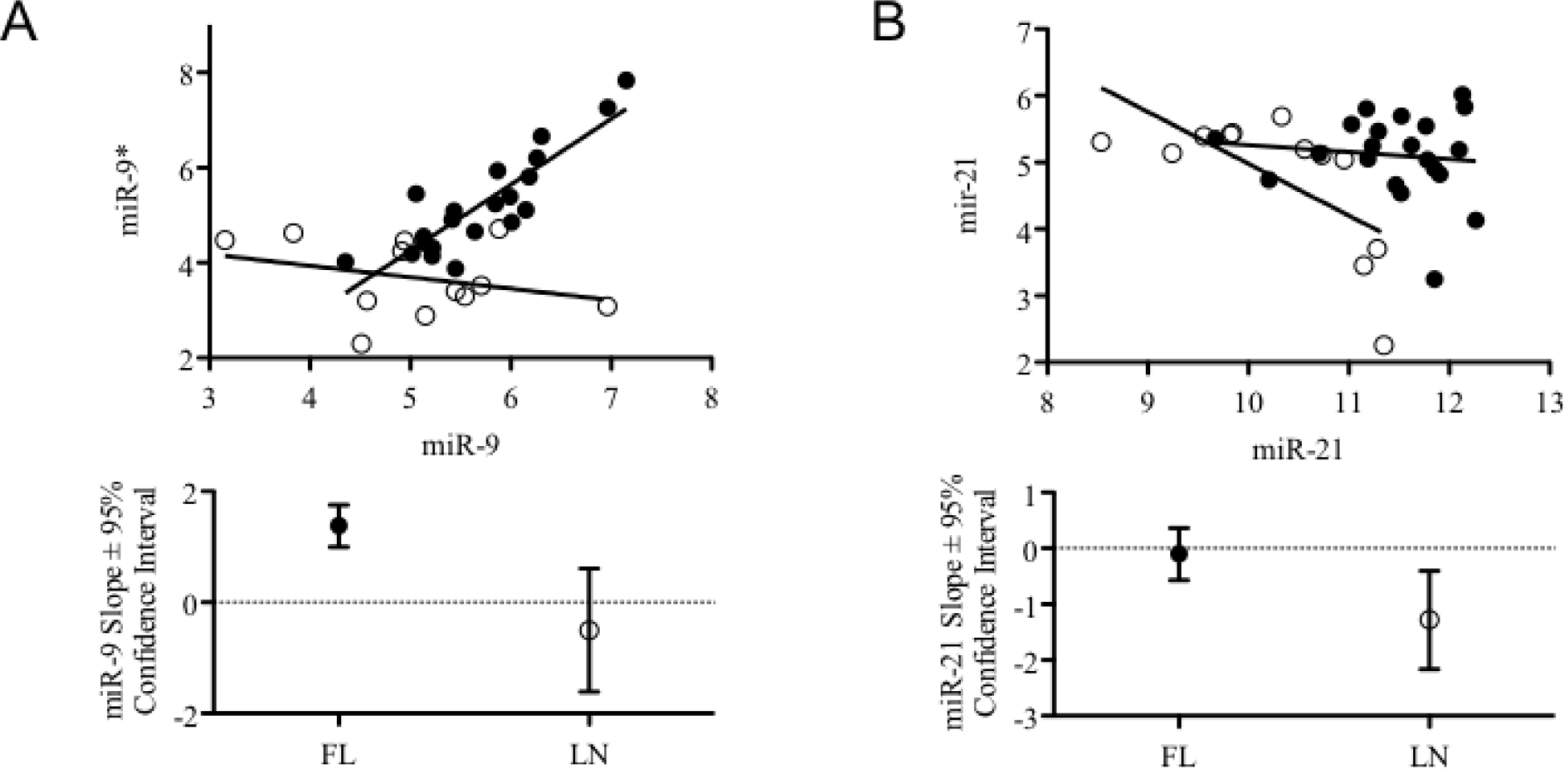
Combined expression levels and trend of 5p and 3p variants of *miR-9-2* and *miR-21* in FLs and rLNs (**A**) Graph reports the expression level of *miR-9-2* and miR-9* (from microarray data) in 21 follicular lymphomas (FLs) cases having clinical information (black circles) and in 12 reactive lymph nodes (rLNs) (empty circles). (**B**) Graph reports the expression level of *miR-21* and miR-21* (from microarray data) in 21 FLs cases having clinical information (black circles) and in 12 rLNs (empty circles). Bottom graphs report slope and 95% confidence interval of the trend lines passing through the symbols of the two series represented in top graphs.

We investigated whether the alteration of miRNA expression shown by FLs could imply coordinated changes of 5p/3p ratios. For most of miRNAs, no difference of 5p/3p relationships between FLs and rLNs could be observed whereas we observed a change of *miR-9-2*/miR-9* and *miR-21*/miR-21* in FLs with respect to rLNs (Figure 3A and 3B). No change of ratio was found for *miR-9-1*/miR-9* and *miR-9-3*/miR-9*.

### T-cells influence the pattern of miRNAs expressed in FLs

**Figure 4:**
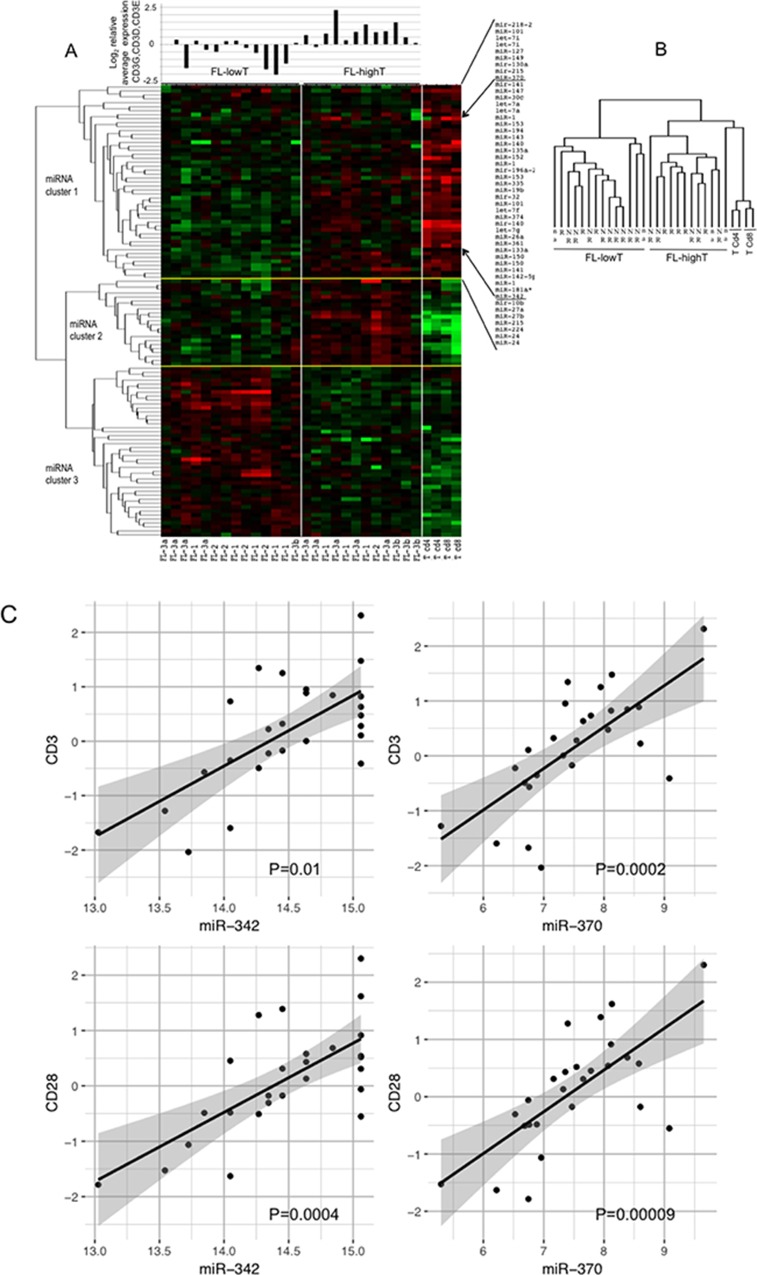
Profile of miRNAs differentially expressed among FLs, CD4^+^ T-cells and CD8^+^ T-cells FL-HT, follicular lymphoma (FL) with high T-cell level. FL-LT, with low T-cell level. (**A**) The heat map describes the expression levels of miRNAs differentially expressed among 30 samples: 26 FLs, two CD4^+^ and two CD8^+^ T-cell samples (FDR 1%). FL-1, FL-2, FL-3a, FL-3b are FLs of grade 1, 2, 3a and 3b, respectively. Red, higher expression (log_2_, +4); green, lower expression (log_2_, -4). On the right of the heat map a partial list of miRNA differentially expressed among the three sets of samples, which are expressed at higher level in T-cells than in FLs (miRNA cluster 1). On the top of the heat map is the average mRNA expression level of three CD3 chains gamma, delta and epsilon for each of the 26 FL samples. Expression data were obtained by qRT-PCR. (**B**) Array tree of 30 samples based on the expression levels of miRNAs differentially expressed. (**C**) Correlation between the expression levels of *miR-342* or *miR-370* (from microrray analysis) and the expression level (log_2_) of *CD3* and *CD28* mRNAs by qRT-PCR in 26 FLs samples. P was calculated by Spearman's correlation.

FL microenvironment is characterized by the presence of a significant number of T-cells, up to 50% of the total cells [25]. To evaluate whether the T-cell component affected the pattern of miRNA expressed in FLs, we compared miRNA expression profiles of FLs with those of two CD4^+^ and two CD8^+^ T-cell samples (Figure 4A and 4B). As expected, FLs and pure T-cells clustered separately and a large number of miRNA discriminated the two categories of samples. Differentially expressed miRNAs split in 3 main clusters. FLs in turn clustered into two groups independently from the FL grade. The same heatmap showing the complete list of differentially expressed miRNAs between FLs and T-cells is reported in Supplementary Figure 2.

We firstly focused on miRNA cluster 1, which included miRNAs expressed at high level in both FLs and T-cells. To evaluate the possible influence of T-cells on miRNA expression profile of FLs, we measured the level of *CD3E*, *CD3D* and *CD3G* mRNA in FLs by qRT-PCR (Figure 4A). The two clusters of FLs were termed FL-LT (low T) and FL-HT (high T) (Figure 4A, miRNAs in cluster 1 listed on the right). Accordingly, by plotting the average expression level of the three *CD3* chains upon each FL we found that the FL-HT group had a significantly higher level of *CD3* than FL-LT (*P* < 0.01) (Figure 4A).

We searched for specific miRNAs representing a possible T-cell signature in FLs. To this aim, we correlated the levels of each miRNA with the average level of *CD3* mRNA. *MiR-342* (*P* = 0.01) and *miR-370* (*P* = 0.0002) showed clear correlation with *CD3* level (Figure 4C). Similarly, correlations were obtained between the mRNA level of *CD28*, another marker of T-cells, and *miR-342* (*P* = 0.0004) or *miR-370* (*P* = 0.0009) (Figure 4C).

Remaining miRNAs differentially expressed between FLs and T-cells discriminated cases belonging to FL-HT and FL-LT. Cluster 2 included miRNAs overexpressed in FL-HT group while cluster 3 included miRNAs overexpressed in FL-LT group (Figure 4A). To gain functional information about the cellular process potentially regulated by differentially expressed miRNAs, we performed pathway enrichment analysis using experimentally validated gene targets of miRNAs (Supplementary Tables 2 and 3). Target genes regulated by cluster 2 were mostly involved in T-cell and B-cell development, in cell cycle, and were under Treg (regulatory T-cells), NF-kB and MYC influence. Main pathways associated with cluster 3 included monocytes, macrophages, dendritic cells, Treg, follicular helper T-cells and B-cell receptor activation, as well as NF-kB, caspase and epithelial-mesenchymal transition.

### Immuno-architectural pattern and total number of Foxp3^+^ and PD1^+^ cells but not CD68^+^ cells discriminate FLs from rLNs

**Figure 5:**
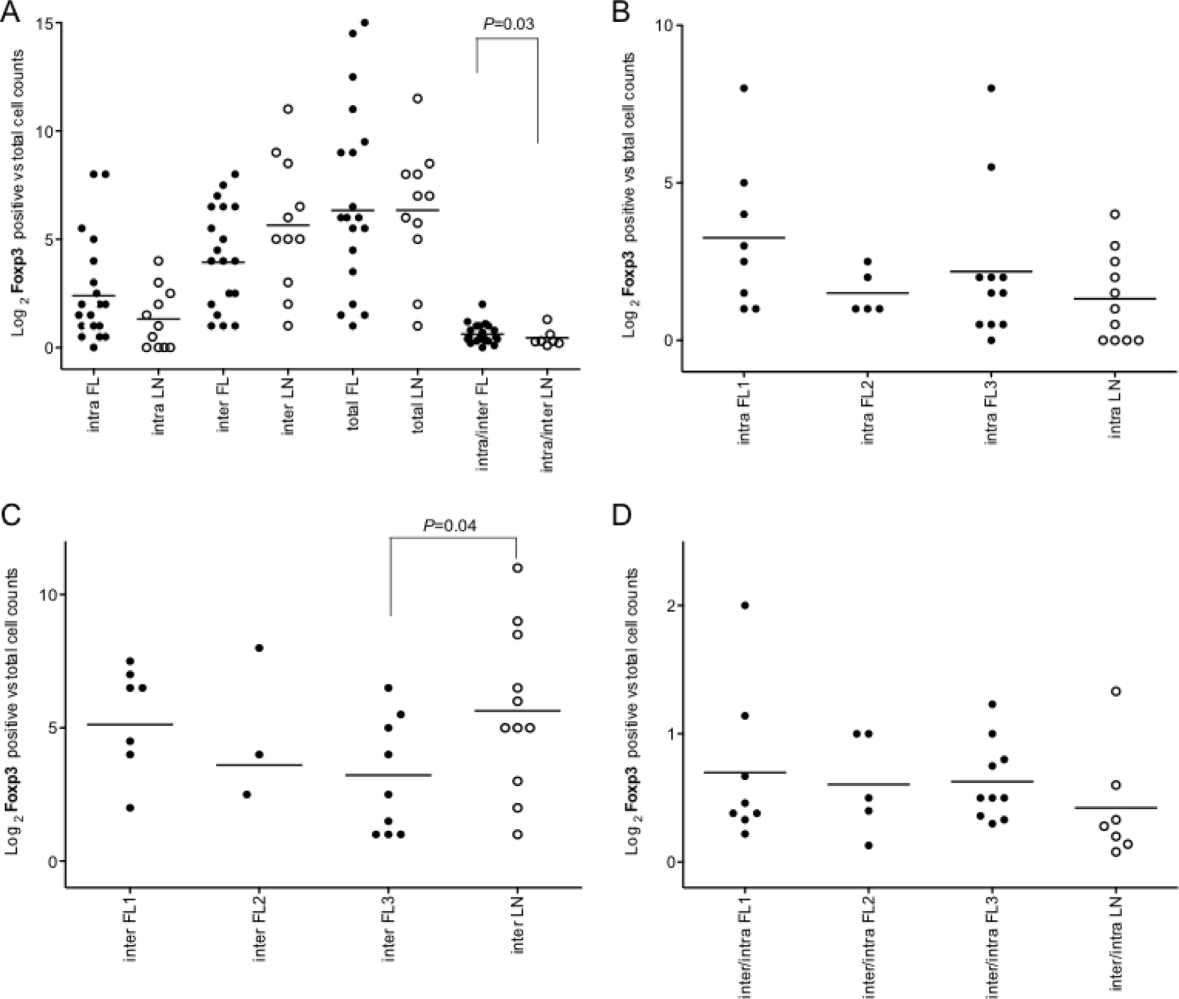
Intrafollicular, interfollicular and total Foxp3^+^ cell count in FLs and rLNs White circles, follicular lymphomas (FLs); black circles, reactive lymph nodes (rLNs); black line, median. Intra, inter and total are intrafollicular, interfollicular and intrafollicular plus interfollicular Foxp3^+^ cells, respectively. Number of Foxp3^+^ cells in each FL and rLN case was the average number of cells in two consecutive slides. The average number of positive cells was normalized on the whole number of nuclei present in a slide. *P* values were calculated by *t*-test. (**A**) Foxp3^+^ cell counts in intrafollicular, interfollicular, total and interfollicular/intrafollicular ratio in FLs and rLN. (**B**) Intrafollicular Foxp3^+^ cell counts in FL grades and LNs. (**C**) Interfollicular Foxp3^+^ cell counts in FL grades and LNs. (**D**) Interfollicular/intrafollicular ratio Foxp3^+^ cell counts in FL grades and LNs.

**Figure 6:**
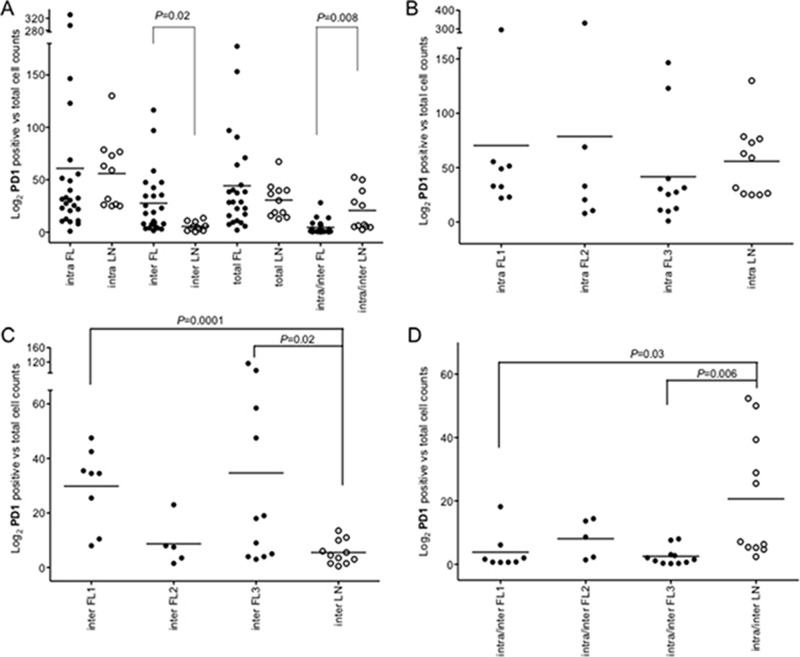
Intrafollicular, interfollicular and total PD1^+^ cell counts in FLs and rLNs White circles, follicular lymphomas (FLs); black circles, reactive lymph nodes (rLNs); black line, median. Intra, inter and total are intrafollicular, interfollicular and intrafollicular plus interfollicular PD1^+^ cells, respectively. Number of PD1^+^ cells in each FL and rLN case was the average number of cells in two consecutive slides. The average number of positive cells was normalized on the whole number of nuclei present in a slide. *P* values were calculated by *t*-test. (**A**) PD1^+^ cell counts in intrafollicular, interfollicular, PD1^+^ total and interfollicular/intrafollicular ratio in FLs and rLNs. (**B**) Intrafollicular PD1^+^cell counts in FL grades and rLNs. (**C**) Interfollicular PD1^+^cell counts in FL grades and rLNs. (**D**) Interfollicular/intrafollicular ratio PD1^+^ cell counts in FL grades and rLNs.

Number and pattern distribution of regulatory T-cells (Foxp3*^+^*), suppressive and exhausted cytotoxic T-cells (PD1*^+^*), and macrophages (CD68*^+^*) were shown to variate considerably among FLs [10, 11, 13]. To evaluate these immune effector subsets in our FL series, we assessed Foxp3^+^, PD1^+^ and CD68^+^ cells by automated IHC in 24 FLs and in 11 rLNs as reference (Figure 5, Figure 6, Supplementary Figures 3–5).

The immuno-architectural structure of immune follicles was taken in account. Only intrafollicular/interfollicular Foxp3^+^ cells ratio was significantly higher in all FLs compared to rLNs (*P* = 0.03) (Figure 5A). Intrafollicular Foxp3^+^ cells were not significantly different in FLs of diverse grade with respect to rLNs (Figure 5B). Interfollicular Foxp3^+^ cells in FL grade 3 were significantly lower in number than in rLNs (*P* = 0.03) (Figure 5C). Intrafollicular/interfollicular Foxp3^+^ cells ratio was not significantly different between FL grade 1, 2 and 3 and rLNs (Figure 5D).

Interfollicular PD1^+^ cells were higher in FLs compared to rLNs (*P* = 0.02) and the intrafollicular/interfollicular ratio was higher in rLNs (*P* = 0.008) (Figure 6A). Intrafollicular PD1^+^ cells were not significantly different in FL grade 1, 2 and 3 compared to rLNs (Figure 6B), while interfollicular PD1^+^ cells were more evident in FL grade 1 (*P* = 0.0001) and FL grade 3 (*P* = 0.02) (Figure 6C). The intrafollicular/interfollicular PD1^+^ cells ratio was lower in grade 1 and grade 3 FL with respect to rLNs (*P* = 0.03 and *P* = 0.006, respectively) (Figure 6D).

In a minority of tumors, high density of macrophages infiltrating the tumor correlated with lower survival [2, 11, 26]. As expected according to morphology, we observed less intrafollicular and more interfollicular CD68^+^ cells in FL compared with rLNs, but both findings were not statistically significant. Total CD68^+^ cells did not change in the two sets (Supplementary Figure 3). A summary of Foxp3^+^, PD1^+^ and CD68^+^ total cell in FLs and rLNs, together with CD3 mRNA levels in FLs, is reported in Supplementary Figure 4. No correlation was found between Foxp3^+^ cells and *CD3* mRNA levels in FLs.

### Foxp3^+^CXCL13^+^ and Foxp3^+^PD1^+^ cells are rare in FL and rLN

In order to characterize the T-cells infiltrating the FL, we investigated the regulatory follicular T cells (TFR) cells, a subset of suppressive T-cells which together with follicular helper T-cells (TFH) cells control the GC reaction [27–29]. TFR cells share phenotypic characteristics with TFH cells and conventional Foxp3^+^ cells and express significant amounts of the prototypic TFH genes Cxcr5, CXCL13, Icos, Bcl6 and PD1 [28, 30]. We assessed slides double stained for Foxp3-CXCL13 as well as Foxp3-PD1 by IHC in FL and rLN samples. Foxp3^+^CXCL13^+^ cells were very rarely observed in FL and rLN (Supplementary Figure 6A and 6B). Foxp3^+^PD1^+^ cells were rare or less than 5% of Foxp3^+^ cells in the neoplastic GC (Supplementary Figure 6C and 6D). A significant number of Foxp3^+^PD1^+^ cells was observed in case T20 (Supplementary Figure 6D). Single-positive CXCL13 or PD1 cells represented a variable fraction mostly in the GC cells (Supplementary Figures 6A–6D).

### CD8^+^ cells are reduced in FL in comparison to rLN

CD8^+^ cells are mainly involved in target cell killing and their increased infiltration was correlated to a better FL prognosis [31]. We assessed abundance and distribution of CD8^+^ cells in FL and rLN (Supplementary Figure 6E and 6F). The frequency of intratumoral CD8^+^ cells ranged from 1.5% to 56.1% of cells in FL patients (Supplementary Figure 7A). CD8^+^ cells were mostly interfollicular and in general less than 2% were intrafollicular. In FL cases T15 and T44, CD8^+^ cells infiltrated significantly also the GC compartment (Supplementary Figure 6E).

CD8^+^ cell counts were lower in FL compared to rLN (*P* = 0.05) (Supplementary Figure 7A). Moreover, CD8^+^ cell counts were not significantly different in relapsed and not relapsed FL. CD8^+^ cells correlated positively with PD1^+^ cells and CD68^+^ cells in FL (either *P* < 0.0001) but not in rLN (Supplementary Figure 7B and 7C).

We calculated the CD8^+^/Foxp3^+^ cell ratio in FL and rLN samples. This ratio did not discriminate FL from rLN and relapsed from not relapsed FLs.

### Twenty-six miRNAs are differentially expressed between relapsed and not relapsed FLs

Twelve patients did not relapse after treatment, while nine FL patients relapsed. We compared the expression levels of miRNAs in relapsed and non-relapsed FLs. According to microarray data, 17 miRNAs were significantly upregulated and nine miRNAs were significantly downregulated in relapsed FLs (*P* < 0.05) (Table 1).

**Table 1:** MiRNAs up-regulated or down-regulated in relapsed versus not relapsed FLs and their reported experimentally validated gene targets

MiRNAs upregulated in relapsed vs not relapsed FL		
miRNA	*P* value	Gene targets^*^
*miR-376c*	0.0046	*ACVR1C, IGF1R, GRB2, TGFBR1, TGFA*
*mir-450-2*	0.0102	-
*mir-431*	0.0110	-
*miR-1*	0.0123	*MCL1, PTEN* and other 17 targets
*miR-382*	0.0126	*BCL6, NFKB* and other 22 targets
*miR-9*	0.0145	*BCL6, ETS, PRDM1* and other 42 targets
*miR-19b*	0.0166	*MYCN, PTEN* and other 21 targets
*miR-522*	0.0190	*SOX2, FXN*
miR-181a^*^	0.0194	*NANOG*
*miR-101*	0.0206	*MYCN, EZH2, FOS, MCL1* and other 26 targets
*miR-320*	0.0264	*MCL1, PTEN* and other 17 targets
*miR-526a*	0.0306	-
*miR-196a*	0.0328	*NFKBIA, BACH1*, 6 HOX genes and other 8 targets
*miR-383*	0.0475	*CCND1, VEGFA* and other 5 targets
*miR-144*	0.0492	*NOTCH1, TGFB1, MTOR, PTEN* and other 10 targets
*miR-184*	0.0500	*BCL2, AKT, MYC* and other 5 targets
miR-9*	0.0500	*RCOR1, ITGB1, GNAI1*
**MiRNAs downregulated in relapsed vs not relapsed FL**		
**miRNA**	***P* value**	Gene targets^*^
*miR-325*	0.0022	-
miR-302c*	0.0072	-
*mir-330*	0.0132	*E2F1, VEGFA, CD44* and other 4 targets
*mir-376b*	0.0149	-
*mir-194-1*	0.0180	*ACVR2B, CDH2, SOCS2* and other 14 targets
*mir-106b*	0.0360	-
*miR-31*	0.0363	*RHOA, PPPR2A, LATS2, SATB2, FOXP3* and other 38 targets
*miR-410*	0.0432	*MET, MDM2*
*mir-491*	0.0494	*BCL2L1, CHD4, TAF10, MMP9, GIT1, SMAD3*

^*^experimentally validated miRNA targets according with miRTarBase, strong evidence, reporter assay [74].

### The expression of ten miRNAs correlates with Foxp3^+^ cell counts in FLs and rLNs

We searched for miRNAs correlating with Foxp3^+^, CD68^+^ or PD1^+^ cells in FLs and rLNs. Ten miRNAs showed correlation with Foxp3^+^ cells in FLs: directly, *mir-325*, *miR-376b*, *mir-450-2* and *miR-515-1* (*P* < 0.05) (Supplementary Figure 8A), and inversely *miR-144*, *miR-302b*, *miR-325*, *mir-431*, *miR-432* and *miR-490* (*P* < 0.05) (Supplementary Figure 8B). For eight of the ten miRNAs correlating with Foxp3^+^ cells in FLs, the relationship between miRNA level and Foxp3^+^ cells was not significantly different in FLs and rLNs. In contrast, the inverse correlation of *miR-144* with Foxp3^+^ cells was lost in rLNs, while the direct correlation of *miR-302b* in FLs turned into inverse correlation in rLNs (*P* = 0.04) (Supplementary Figure 8A and 8B).

No correlation was observed between total CD68^+^ or PD1^+^ cells and miRNAs level in FLs and rLNs.

### Foxp3^+^ cell counts are lower in relapsed FL

**Figure 7:**
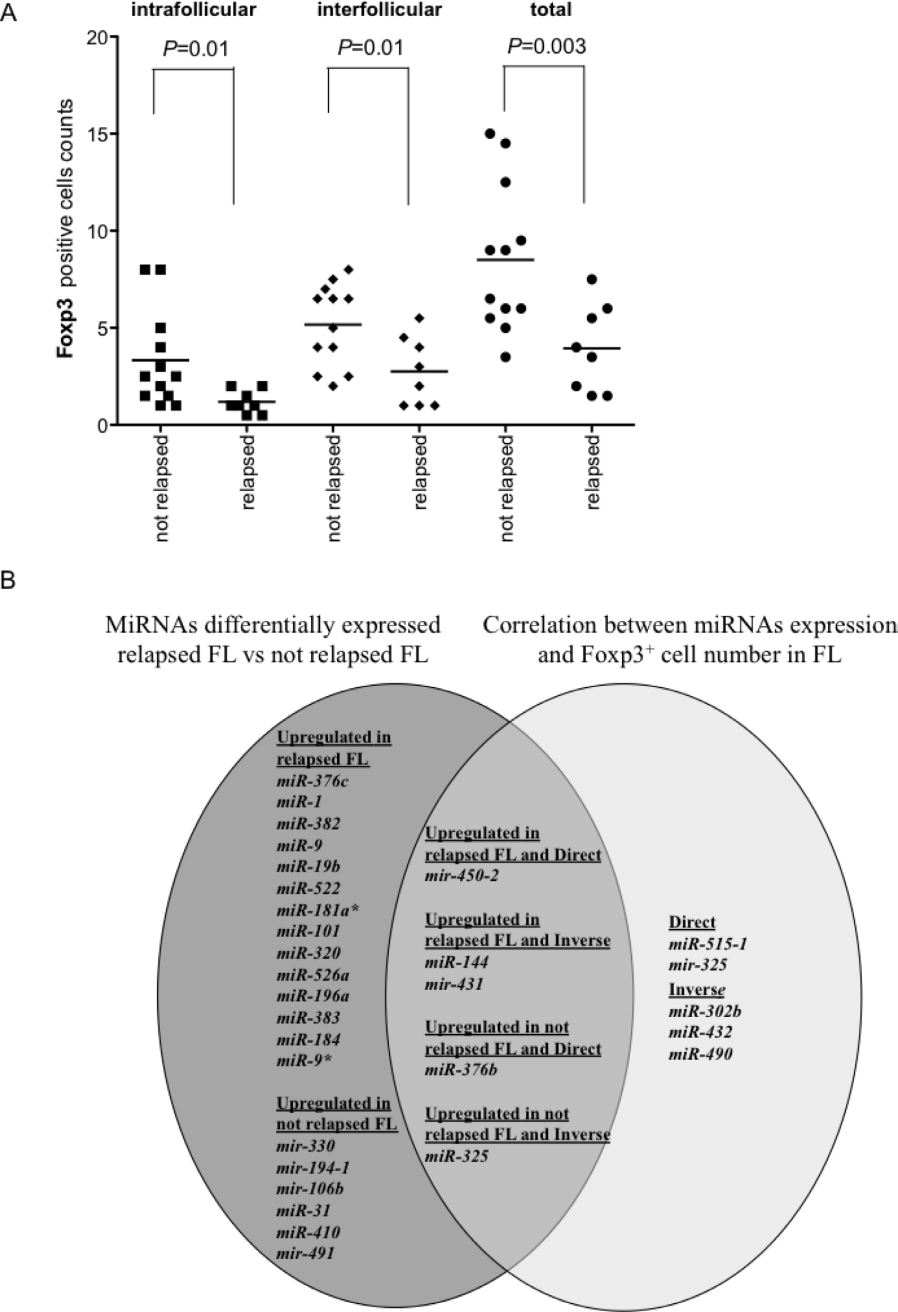
Foxp3^+^ cell counts in relapsed and not relapsed FLs and Venn diagram of miRNAs upregulated in relapsed or not relapsed FLs and of miRNAs correlated directly or inversely with Foxp3^+^ cell counts Number of Foxp3^+^ cells in each follicular lymphoma (FL) was the average of cells in two consecutive slides. The average number of positive cells was normalized on the whole number of nuclei present in a slide. (**A**) Comparison of intrafollicular, interfollicular and total Foxp3^+^ cells in relapsed and not relapsed FLs. *P* values were calculated by *t*-test. (**B**) Venn diagram of miRNAs upregulated in relapsed or not relapsed FLs (dark grey) and the intersection (intermediate grey) with miRNAs correlated directly or inversely with Foxp3^+^ cell counts (light grey).

Clinico-pathological characteristics of FL patients were described in Table 2. Foxp3^+^, PD1^+^ and CD68^+^ cells were compared in relapsed and non-relapsed FLs. PD1^+^ and CD68^+^ cells were not significantly different between relapsed and non-relapsed FLs (*P* > 0.1). Instead, intrafollicular, interfollicular and total Foxp3^+^ cells were less represented in relapsed than in non-relapsed FLs (*P* = 0.01, *P* = 0.01, *P* = 0.003, respectively) (Figure 7A).

**Table 2:** FL patients, drug treatments, IHC markers and clinical status

FL	Grade	Age	FLIPI	Treatment	IHC Bcl2	IHC Bcl6	Clinical status
T15	1	52	Low	No treatment	2	2	NR
T16	1	41	Low	CHOP	2	1	NR
T17	1	50	Low	RT	2	1	R
T18	1	48	Intermediate	DHAP	2	1	R
T19	1	47	Intermediate	CHOP + DHAP	2	1	NR
T20	1	76	High	FluCy	0	2	NR
T21	1	51	Intermediate	APO + DHAP	1	1	NR
T22	1	47	Low	CHOP	1	1	NR
T23	2	37	Intermediate	CHOP + DHAP	2	2	NR
T24	2	65	High	CHOP	2	1	R
T25	2	60	High	CHOP + DHAP	1	1	NR
T26	2	55	Intermediate	CHOP	2	1	NR
T28	2	65	High	CHOP + DHAP	2	1	NR
T30	3a	70	High	CHOP + RT	1	1	NR
T31	3a	64	High	CLB + CHOP	0	2	R
T33	3a	n.a.	n.a.	n.a.	2	1	n.a.
T34	3a	71	High	CLB	1	1	R
T35	3a	69	High	CHOP	2	1	NR
T37	3a	56	Low	Surgery	2	1	R
T38	3a	47	Low	CHOP	1	1	R
T39	3a	56	Intermediate	APO + DHAP	2	1	NR
T40	3a	67	Low	RT	2	1	R
T42	3b	n.a.	n.a.	n.a.	1	1	n.a.
T43	3b	70	High	Steroids	1	1	n.a.
T44	3b	n.a.	n.a.	n.a.	1	1	n.a.
T45	3b	77	Intermediate	CLB	0	1	NR

n.a., not available; R, relapsed; NR, not relapsed. IHC: 0, absent; 1, 1+, weak stain; 2, 2+ strong stain; CHOP: cyclophosphamide, adriamycin, vincristine and prednisone-based regimen; APO: adriamycin, vincristine and prednisone-based regimen; DHAP: dexametasone, high dose-cytarabin and cisplatin-based regimen; FluCy: fludarabin and cyclophosphamide-based regimen; CLB: clorambucil; RT: radiotherapy.

The comparison between miRNAs involved in FL relapse and miRNAs correlating with Foxp3^+^ cell counts in FLs was summarized by Venn diagram (Figure 7B). Five miRNAs intertwined the two pools. *Mir-450-2* was upregulated in relapsed FLs and correlated positively with Foxp3^+^ cells. *MiR-144* and *mir-431* were upregulated in relapsed FLs and correlated inversely with Foxp3^+^ cells. *MiR-376b* was upregulated in not relapsed FLs and correlated directly with Foxp3^+^ cells. Finally, *MiR-325* was upregulated in not relapsed FL and correlated inversely with Foxp3^+^ cells.

### Foxp3^+^ cell counts associate with shorter time to relapse

**Figure 8:**
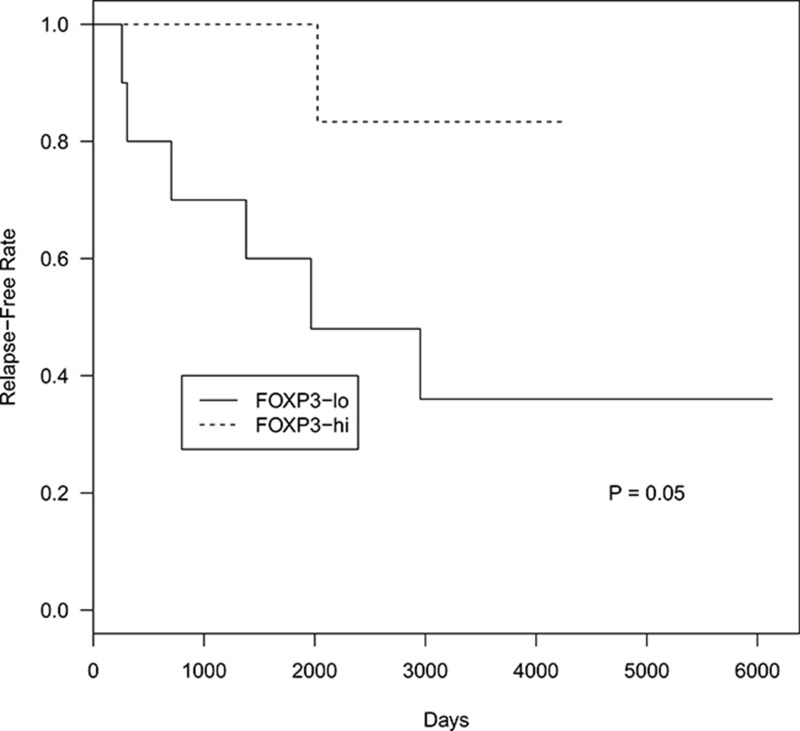
Time to relapse probability curves based on total Foxp3^+^ cell counts in FL patients Top curve represents FL cases with total Foxp3^*+*^ cells higher than median. Bottom curve represents FL cases with total Foxp3^*+*^ cells lower than median.

PD1^+^ and CD68^+^ cells did not associate with time to relapse in FLs. Regarding Foxp3^+^ cells, FLs were subdivided in two categories according to the Foxp3^+^ cells, higher and lower than the median. FLs with lower Foxp3^+^ cells associated with a shorter time to relapse (*P* = 0.05) (Figure 8), with a median time to relapse of 2000 days.

## DISCUSSION

The miRNA network supervises the cell proteome during lymphopoiesis and turned out essential in lymphomagenesis [32–34]. Although the importance of miRNA functions in the regulation of cellular processes is clear, both miRNAs deregulation and its possible use as a biomarker in FLs have been only partially addressed.

The tumor microenvironment provides supportive signaling for the proliferation of FL cells, as well as protection against attack from the immune system [12, 35, 36]. Several results have drawn attention to the host immune response in FLs [12]. FLs harbor up to 50% of T-cells [25]. Altered ratios between B-cells and other immune cells in FLs compared to rLNs would drive evident modifications of the level of molecules (i.e. mRNA and miRNAs) shared with lymphoma cells. Accordingly, two different gene expression signatures, which correlated with different outcomes, were identified in FLs [8]. A group included long surviving FLs expressing T-cell-derived genes; another group included FLs with worse prognosis and enriched of genes typical of dendritic cells and macrophages.

The molecular heterogeneity observed in our FLs and rLNs series by miRNA profiling (Figure 1) was clear in previous studies reporting the comparison of miRNA expression profiles in FLs and rLNs [21, 37, 38] but not in another study [23]. To test the hypothesis that the miRNA profile of FLs implies an immune signature, we compared FLs and pure T-cells, more abundant among the immune cells infiltrating the FLs. Similarly to what observed by gene expression data, FLs split into two clusters. The FL-HT cluster included miRNAs expressed at higher level in both FLs and T-cells (miRNA cluster 1). The higher level of *CD3* mRNA in this group of FLs point to T-cells as main driver of this signature. These findings suggest that the clonal expansion of abnormal B-cells alters the immune response microenvironment, leaving a trace in the miRNA profile of FLs.

Being expressed at higher level in FL than in pure T-cells, both the cluster 2 miRNAs (overexpressed in FL-HT group) and the cluster 3 miRNAs (overexpressed in FL-LT group) could not be ascribed to a T-cell signature. Instead, miRNAs of the cluster 2 (mainly members of the tricistrons *miR-199b*/*100-125b*) were found expressed at higher level in NK cells, some myeloid cell lineages and in CD4^+^ rather than CD8^+^ T-cells [39]. Tumor cells suppress the antitumor immune response from helper T-cells, cytotoxic T lymphocytes, and macrophages by various mechanisms [40]. Consequently, the feature signatures of the two FL groups might recapitulate how the malignant B-cells alter the function of T-cells in the FL microenvironment in order to escape immunosurveillance.

Pathway enrichment analysis on target genes of miRNA cluster 2 showed a significant enrichment in pathways related to B-cells and T-cells activation and development as well as to cell cycle and control by MYC. For miRNA cluster 3, we found enrichment in pathways connected with mononocytes, macrophages, T helper and B-cell activation, as well as NF-kB, UV response and apoptosis. These results are reminiscent of the identification of two groups of FLs with different prognosis based on the gene expression signatures [8].

The two members of the cluster 1 *miR-342* and *miR-370* strongly correlated with two representative markers of T-cells *CD3* and *CD28*. A connection between *miR-342* and T-cells infiltrating the lymphomas was previously reported. *Mir-342* was reported to be higher in T-cells than B-cells and in naive and Th17 CD4^+^ than in CD8^+^ T-cells [41, 42]. Moreover, *MiR-342-3p* expression associated the CD3^+^ T-cells in ALK^-^ anaplastic large cell lymphoma [43]. Since B-cell lymphomas may display an increased CD4^+^/CD8^+^ T-cells ratio with respect to rLNs, it is possible that in FLs *miR-342* represents a signature associated with CD4^+^ T-cells [35]. Abnormal number and activity of Th17 cells has been associated to autoimmune disorders and inflammation [44]. In B-cell lymphomas, altered B-cells might skew the balance of Treg cells and Th17 cells versus the increase of inhibitory microenvironment [45].

Various miRNAs reported to be differentially expressed in FLs play regulatory roles in lymphomagenesis [46]. We identified a set of 27 miRNAs differentially expressed between FLs and rLNs. Among these, miR-9*, *miR-16*, *miR-195*, *miR-15b* and *miR-374*, but not *miR-30-5p*, were previously found overexpressed in FL compared to rLNs [23]. Moreover, miR-9* and *miR-155* have been found overexpressed also in B-cells purified from FLs with respect to those from rLNs [24]. Among 17 miRNAs upregulated in FLs, *miR-29* family, *miR-195*, *miR-15/16*, *miR-30* family, *miR-146a* are known to be downregulated as a consequence of MYC activity on their promoters [47]. Most of miRNAs upregulated and downregulated in FLs are equally modulated in germinal centre B-cells in comparison to naive B-cells (our unpublished data).

We identified a subset of seven miRNAs which discriminated grade 1 from grade 3a FLs. These data represent the first description of an increased alteration of miRNAs from low to high FL grade. The direct comparison between FLs of different grade did not return significant results, possibly in consequence of the low number of cases and the heterogeneity previously cited. Among the miRNAs altered in grade 3, *miR-320* downregulation could be related to the reported increase of its gene target *MCL1*, which strongly associated with FL onset [48, 49].

MiRNA biogenesis is a complex process regulated at multiple stages [18]. The mechanisms responsible for the alteration of miRNA processing are not well known, in particular in lymphomas. Among all miRNAs here examined for the proportion 5p/3p, we found that *miR-9-2*/miR-9* and *miR-21*/miR-21* proportions changed in FLs with respect to rLNs. The peculiarity of *miR-9* in FL was previously noticed. In fact, particularly high interindividual differences of *MIRN9* and MIRN9* in FL was observed [23]. *MiR-9* was also one of the 26 miRNAs which showed an altered proportion between different miRNA forms in primary effusion lymphoma [50]. Since *miR-9-*2 gene was reported to be methylated in normal CD19^+^ B-cells [51], the lymphomagenesis could imply changes of *miR-9-*2 methylation and of its expression. It is not yet demonstrated whether *miR-9* alteration point to a etiologic field defect for FL genesis [52].

The level of specific miRNAs associated with clinical behaviour in our cohort of FL patients [23]. We could not associate miRNA expression and overall survival due to the low number of deaths in our series. Instead, we identified a set of 26 miRNA differentially expressed between relapsed and non-relapsed FL. None of the 26 miRNAs overlapped with those reported in the literature to be associated with FL outcome. Among miRNAs discriminating relapsed from non-relapsed FLs, several have been linked to drug resistance in lymphomas and other cancer types.

Malignant B-cells can attract Tregs or suppressive cells, thus influencing the patterns of miRNA expressed in FL tissues. Foxp3^+^ Tregs are relevant for B-cell development as they control the germinal centre response [28]. We observed a direct correlation of four miRNAs and an inverse correlation of six miRNAs with total Foxp3^+^ cells in FLs. Notably, miRNAs level and PD1^+^ or CD68^+^ cells did not show any correlation, thus supporting the idea that Foxp3^+^ cells are those mainly involved in setting of miRNAs level. *MiR-144* and *miR-302b* level, which correlated inversely with Foxp3^+^ cells in FL, lost this trend in rLNs. *MiR-144* was shown to target *BCL6* in diffuse large B-cell lymphoma [53]. Since relapsed FLs showed higher level of *miR-144* and lower number of Foxp3^+^ cells, it can be hypothesized that both events might cooperate in supporting the proliferation of FL cells.

In FL, Foxp3^+^ Treg cells might represent from less than 5% up to 25% of the germinal center T-cells [10, 28]. Treg cells might have significant clinical relevance in FL since they can serve as tumor-killing cells. Lower number of Tregs correlated with refractory disease, transformation, and aggressive histology of FL [10, 14, 54]. However, other studies failed to confirm this conclusion [55, 56]. Rather than Treg cells total content, the architectural distribution and pattern of Foxp3^+^ cells correlated with the outcome of patients [55]. Intrafollicular Foxp3^+^ cells pattern, but not a diffuse pattern, was found associated with poorer overall survival in FL patients. Our data confirmed the clinical relevance of both total number and pattern of Foxp3^+^ cells in FL patients. In our FL series, intrafollicular, interfollicular and total Foxp3^+^ cells were lower in relapsed than not relapsed FLs. Moreover, interfollicular Foxp3^+^ cells in grade 3 FLs, but not in FLs of inferior grade, were lower than in rLNs. Importantly, lower than median Foxp3^+^ cells associated with shorter time to relapse of FL patients. Taken together, our results and those reported by Farinha *et al.* [55] suggest that the immuno-architecture of the microenvironment around transformed B-cells impacts on patient outcome in terms of time to relapse and of survival of FL patients.

PD1 receptor was demonstrated to suppress cytokines signaling in lymphoma-infiltrating T-cells [57]. PD1 is expressed by intratumoral CD4^+^ follicular helper T-cells and from exhausted cytotoxic T-cells [58]. A diffuse pattern of PD1 staining associated with a shorter time before transformation and inferior overall survival [13, 58]. At variance with these authors, according to our data no association was established between the pattern of PD1 positivity and time of relapse.

TFH cells and TFR cells subtypes are recognized essential for the physiological regulation of humoral immunity. TFR cells are immune suppressive by limiting TFH cells action and GC B-cells number [28, 59, 60]. The specification of the two T-cell populations is driven by mechanisms operated by lineage-specific transcription factor as T-bet and BCL6 [61] and by mesenchymal stem cells [62]. It is believed that TFR cells arise from thymic regulatory T-cells. However, absence of Foxp3 expression in GC suppressive T-cells suggested also a possible origin of TFR from TFH cells [63]. TFR cells were reported to express significant amounts of the prototypic TFH genes CXCR5, CXCL13, ICOS, BCL6 and PD1 in mouse [28]. Foxp3^+^PD1^+^CXCL13^+^ TFR cells were detected in various mice and human settings [27–29, 64, 65]. Foxp3^+^PD1^+^ cells have been previously reported to be rare or absent in human FL, diffuse large B-cell lymphoma, Hodgkin's disease, T-cell lymphoma normal tonsils and rLN [54, 66–69]. Small amount (1–4%) or absence of Foxp3^+^PD1^+^ cells were found in breast cancer tissues [70, 71], suggesting the possibility that the phenotype of TFR cells in lymphomas and cancer does not overlap those in other physiological contexts [62]. We observed clear staining for PD1 or CXCL13 in sparse cells within the neoplastic follicles of FL and the two signals were almost always independent from that of Foxp3. Only one FL cases showed a significant number of double stained cells. The general rarity of TFR cells in FL and rLN could be suggestive of a skewed T-cell differentiation in sustaining the immune activation.

The recruitment of CD8^+^ cells into the lymph nodes was found protective of a disease-free survival in FL patients [25]. We found that CD8^+^ cells distributed mainly in interfollicular areas and were in higher number in FL than in rLN. In our series CD8^+^ cell counts did not associate with relapse and not relapsed status of FL patients.

In conclusion, our data reveal new miRNAs involved in FL in comparison with rLN. The miRNA signature in FL was interpreted in light of the significant infiltration of immune cells typical of a quote of FLs. In this regard, lower infiltration by Foxp3^+^ cells is a feature of relapsed FLs and also associated with shorter time to relapse. High levels of *miR-144* and *mir-431*, which are upregulated in relapsed FL and inversely correlated with Foxp3^+^ cell number, and of *miR-376b,* which is upregulated in non-relapsed FL and is directly correlated with Foxp3^+^ cells, might complement low Foxp3^+^ cell counts as a relapse risk predictor in FL patients. Multivariate analysis to assess combination of Foxp3^+^ cell number and *miR-144*, *mir-431, miR-376b* levels as markers of FL relapse was not possible in this context owing to the low number of events. The diagnostics implication of the combined measure of miRNAs and Foxp3 for the risk of relapse needs to be confirmed on a larger FL cohort.

## MATERIALS AND METHODS

### Samples

FL samples were obtained from patients accessing care in the Hematology section of the University of Verona Hospital compound (Table 1). All biopsy and blood samples were obtained after informed consent approved by Ethical Committee of AOUI of Verona (N. 1828, May 12, 2010 “Institution of cell and tissue collection for biomedical research in Onco-Hematology”). Twenty-six patients with FL grade 1 or 2 or 3 (subdivided in 3a and 3b) for whom fresh/frozen pre-treatment samples were available were included. Twenty-one patients were treated with first-line chemotherapy (cyclophosphamide or doxorubicin or etoposide or prednisone or clorambucil), radiotherapy or surgery. One patient received no chemotherapy and another one only radiotherapy. Clinical follow-up was available for 21 FL patients. The average follow-up of patients was of 13.5 years. Time to relapse was defined as the time interval between complete remission achievement and relapse. Control samples were 12 lymph nodes (rLNs) characterized by reactive follicular hyperplasia. CD4^+^ and CD8^+^ T-cells were isolated from blood of two healthy donors by Ficoll-Paque (Pharmacia, Germany) density gradient centrifugation, followed by positive immune affinity magnetic sorting.

### Immunohistochemistry (IHC)

Number of Foxp3^+^, PD1^+^, CD68^+^ and CD8^+^ cells were quantified in whole-tissue sections of all samples using an automated scanning microscope and image analysis system (S.CORE Web Based Image Analysis, S.CO LifeScience, Germany). Double staining for Foxp3-PD1 and Foxp3-CXCL13 was performed using an automated stainer (Leica BondMax) following standard protocols, as previously described [72]. Ab for Foxp3 was revealed by horsereadish peroxidase (nuclear). The slides were incubated with a second Ab then revealed by alcaline phosphatase (cytoplasmic). For each slide, “intrafollicular”, “interfollicular” and total positive cells were determined. The number of positive cells in each FL and rLN sample was the average number of cells in two consecutive slides. The average number of positive cells used for subsequent analysis was normalized on the whole number of nuclei present in a slide.

### Microarrays

MiRNAs labeling and hybridization were performed using 5 μg total RNA, as described [73]. MiRNAs were named according with the old nomenclature reported in miRBase (www.mirbase.org). MiR-X was the 5p or 3p form of a miRNA and mir-X is the 5p or 3p remaining form of MiR-X.

### Quantitative RT-PCR

Validation of differentially expressed miRNAs and mRNA quantification was performed by quantitative RT-PCR as described in Malpeli *et al.* [34]. Taqman assays and oligonucleotide primers used are listed in Supplementary Table 1.

### Data analysis

Microarrays data analyses were performed as described in Malpeli *et al.* [34]. Microarray data sets are available on the ArrayExpress under http://www.ebi.ac.uk/arrayexpress/experiments/E-MTAB-5844. To select significant differences among expressed gene or categories of samples, we performed using Anova tests. Correlation coefficients between expression of 5p and 3p miRNA forms were calculated using Deming linear regression. Pathway analysis on predicted miRNA targets was performed by Gene Set Enrichment Analysis (GSEA). Experimentally validated miRNA targets (strong evidences) taken from MirTarBase at http://mirtarbase.mbc.nctu.edu.tw [74] were submitted to GSEA http://software.broadinstitute.org/gsea/msigdb/index.jsp [75, 76]. More significant Immunological Signatures and Hallmark Gene Sets were considered.

A more extensive description of the methods for Nucleic acids, Quantitative RT-PCR, Immunohistochemistry, Microarrays and Data Analysis can be found in Supplementary Material.

## SUPPLEMENTARY MATERIALS TABLES AND FIGURES



## Abbreviations

FL: follicular lymphoma
IHC: immunohistochemistry
rLN: reactive lymph node
FDR: false discovery rate
FL-HT: follicular lymphoma high T-cells
FL-LT: follicular lymphoma low T-cells
Treg: regulatory T-cells
TFR: regulatory follicular T-cells
TFH: follicular helper T-cells
