# Supplementary material for: MicroRNA signatures and Foxp3+ cell count correlate with relapse occurrence in follicular lymphoma
Source: Oncotarget. 2018 Apr 13;9(28):19961–79. doi: 10.18632/oncotarget.24987 (PMC5929439; doi:10.18632/oncotarget.24987)
Supplement: Supplementary file 1 [file oncotarget-09-19961-s001.pdf]

MicroRNA signatures and Foxp3+ cell count correlate with relapse occurrence in follicular lymphoma

SUPPLEMENTARY MATERIALS

Nucleic acids

Five µm slides of FL and rLN frozen tissues were cut at the cryostat. Tumor cellularity of FL, as assessed by an expert pathologist, was higher than 70%. Total RNA was isolated from frozen tissues and sorted cells using TRIZOL reagent (Invitrogen) according to manufacturer protocol. RNA concentration and integrity was determined respectively by spectrophotometer and agarose gel separation.

Quantitative RT-PCR (qRT-PCR)

For each sample and miRNA, 5 ng of total RNA was converted to cDNA by TaqMan MicroRNA Reverse Transcription kit (Applied Biosystems) using the miRNA specific primer contained in the TaqMan MicroRNA assays (Applied Biosystems). MiRNA expression was evaluated in 1× TaqMan Universal Master Mix (Applied Biosystems). For mRNA expression analysis, 1 µg of total RNA was converted to cDNA using the cDNA Universal Reverse Transcription Kit (Applied Biosystems). MRNA expression was evaluated in 1x Power Sybr Green Master Mix (Applied Biosystems). TaqMan miRNA assays and oligonucleotide primers used in qRT-PCR analysis are listed in Supplementary Table 1. QRT-PCR analysis was performed by 7900HT SDS instrument (Applied Biosystems). Expression differences among samples were determined by the comparative method according to User Bulletin #2 (Applied Biosystems) using the average level of noncoding RNA *RNU44* and *U47* for miRNAs and *GAPDH* for mRNAs as reference.

Immunohistochemistry (IHC)

Number of $Foxp3^+$ (mAb 221D/D3, Serotec, 1:200) $PD1^+$ (mAb NAT105, UCS Diagnostics, 1:100), $CD68^+$ (mAb KP1, DAKO, 1:50) and $CD8^+$ (mAb C8/144B, DAKO, 1:200) cells were quantified in whole-tissue sections using an automated scanning microscope and image analysis system (S.CORE Web Based Image Analysis, S.CO LifeScience, Germany). For double staining Foxp3-PD1 and Foxp3-CXCL13, Foxp3 was revealed by horsereadish peroxidase and the second antibody revealed by alkaline phosphatase. CXCL13 polyclonal antibody (RD Systems) was diluted 1:50. A hematoxylin and eosin stain was prepared using routine methods. Immunostaining was performed using a Dako autostainer. The number of positive cells in each FL and rLN case was the average of cells counted in two consecutive slides. The average number of positive cells used for subsequent analysis was normalized on the whole number of nuclei present in a slide. Immunoarchitectural distribution of the different markers was determined in relation to the neoplastic follicles. For each slide, "intrafollicular" and "interfollicular" and total positive cells were determined.

Microarrays

MicroRNA labeling and hybridization were performed using 5 µg total RNA, as described by Liu G *et al*., PNAS, 2008**.** We used a multi-species microarray platform containing 2,284 probes, 1,256 for human and 1,028 for mouse targets, respectively. A total of 353 human mature or pre- miRNAs were detectable by the microarray. Each human target was matched by at least two probes, with an average of 4.3 probes for each target. Hybridization signals were detected with Streptavidin-Alexa647 conjugate and scanned images (Axon 4000B) were quantified using the Genepix 6.0 software (Axon Instruments). To minimize the possible batch effect on miRNAs expression, samples of the same category were randomized through different batches. MiRNAs were named according with the old nomenclature reported in miRBase (www.mirbase.org). MiR-X was the 5p or 3p form of a miRNA according to the old nomenclature and mir-X is the 5p or 3p remaining form of MiR-X.

Data analysis

Expression data from microarrays were normalized and transformed using the *vsn* package for R. Microarray data sets are available on the ArrayExpress under http://www.ebi.ac.uk/arrayexpress/experiments/E-MTAB-5844. The spots were subsequently classified based on their target sequence regardless of the original designation. The expression measures for probes matching the same miRNA sequence were summarized using the *medpolish*

algorithm, in order to obtain a unique expression figure for each target. Clustering analysis was performed using the *hclust* function and the inverse Pearson correlation as a distance metric, for both genes and arrays. All the clusters were visualized using the Java TreeView software (http://jtreeview.sourceforge.net). To select differentially expressed genes, we performed either Anova or *t*-tests. To take into account multiple hypothesis testing, the FDR (false discovery rate) was calculated using the *q-value* package for R. All the calculations were performed using the R statistical software (http://www.r-project.org). Correlation was calculated using Deming linear regression. Pathway analysis was performed by Gene Set Enrichment Analysis. Experimentally validated, strong evidences, miRNA targets were taken from MiRTarBase at http://mirtarbase.mbc.nctu.edu.tw and were submitted to http://software.broadinstitute.org/gsea/msigdb/index.jsp. More significant Immunological Signatures and Hallmark Gene Sets were considered.

Supplementary Table 1: TaqMan miRNA assays or oligonucleotide primers used for quantitative RT-PCR

Gene	TaqMan miRNA assays or oligonucleotide primers
let-7i	4395332
miR-9	4373285
miR-9*	4395342
miR-21	4373090
miR-28	4373067
miR-195	4373105
miR-219	4363080
miR-320	4395388
miR-340	4395369
miR-342	4373040
miR-370	4427975
RNU44	4380911
U47	4373384
CD28	F-GAACCATTATCCATGTGAAAGGG<br>R-TAGAAGGTCCGGGAAATAGGG
CD3D	F-CCTTCTCTCGCAAGTGAGCC<br>R-CCGTTCCCTCTACCCATGTG
CD3E	F-CAAGCCTGTGACACGAGGAG<br>R-TCCGGATGGGCTCATAGTCT
CD3G	F-TCTTTGCTGAAATCGTCAGCA<br>R-GCTTGTCTGAAGCTCTCGACTG
GAPDH	F-ATCAGCAATGCCTCCTGCAC<br>R-TGGTCATGAGTCCTTCCACG

Supplementary Table 2: Pathway enrichment using experimentally validated gene targets of miRNA cluster 2*

Gene Set Name	Pathway Description	FDR *Q* value
**Immunologic Signatures**		
GSE9006	Genes up-regulated in comparison of peripheral blood mononuclear cells (PBMC) from healthy donors versus PBMCs from patients with type 1 diabetes at 4 month after the diagnosis.	3,00E-29
GSE2405	Genes down-regulated in polymorphonuclear leukocytes (9h): control versus infection by A. phagocytophilum.	1.54E-28
GSE22025	Genes up-regulated in CD4 T cells: progesterone versus TGFB1 and progesterone.	4.71E-26
GSE42021	Genes up-regulated in T reg: peripheral lymph nodes versus thymic CD24 int.	4.71E-26
GSE6674	Genes down-regulated in B lymphocytes: anti IgM versus PL2-3 (Chromatin IC).	4.71E-26
GSE9006	Genes up-regulated in comparison of peripheral blood mononuclear cells (PBMC) from healthy donors versus PBMCs from patients with type 1 diabetes at 1 month after the diagnosis.	4.71E-26
GSE27434	Genes down-regulated in T reg: wild type versus DNMT1 knockout.	3.26E-25
GSE41978	Genes down-regulated in CD8 T effector cells during infection: KLRG1 high versus KLRG1 low.	3.26E-25
GSE42021	Genes down-regulated in T conv from: peripheral lymph nodes versus thymic precursors.	2.61E-24
GSE369	Genes up-regulated in liver from SOCS3 knockout: untreated versus IL6 injection.	2.05E-23
GSE339	Genes down-regulated in comparison of CD8 dendritic cells (DC) versus CD4- CD8- DCs.	1.25E-22
GSE42021	Genes up-regulated in T reg: peripheral lymph nodes versus thymic CD24 high.	1.25E-22
GSE4748	Genes up-regulated in monocyte-derived dendritic cells: untreated versus LPS (3h).	1.25E-22
GSE5589	Genes up-regulated in bone marrow-derived macrophages with IL6 knockout and 45 min of stimulation by: LPS versus IL10 and LPS.	1.25E-22
GSE10239	Genes down-regulated in comparison of naive vs effector CD8 T cells (4-5 days postinfection).	7.24E-22
GSE10239	Genes up-regulated in comparison of naive CD8 T cells versus memory CD8 T cells.	7.24E-22
GSE2405	Genes up-regulated in polymorphonuclear leukocytes (24h): control versus infection by A. phagocytophilum.	7.24E-22
GSE37533	Genes down-regulated in CD4 T cells treated with pioglitazone and over-expressing: FOXP3 and PPARg1 isoform of PPARG versus FOXP3.	7.24E-22
GSE41867	Genes down-regulated in CD8 T cells: naive versus effectors at day 8.	7.24E-22
KAECH NAIVE VS DAY8 EFF CD8 TCELL UP	Genes up-regulated in comparison of naive versus effector CD8 T cells at the peak expansion phase (day8 after LCMV-Armstrong infection).	7.24E-22
**Hallmark Gene Sets**		
G2M CHECKPOINT	Genes involved in the G2/M checkpoint, as in progression through the cell division cycle.	1.89E-36
MYC TARGETS	A subgroup of genes regulated by MYC - version 1 (v1).	1.54E-31
E2F TARGETS	Genes encoding cell cycle related targets of E2F transcription factors.	8.03E-26
UV RESPONSE_DN	Genes down-regulated in response to ultraviolet (UV) radiation.	9.83E-25
TNFA SIGNALING VIA NFKB	Genes regulated by NF-kB in response to TNF.	3.59E-24
MITOTIC SPINDLE	Genes important for mitotic spindle assembly.	2.48E-23
EPITHELIAL MESENCHYMAL TRANSITION	Genes defining epithelial-mesenchymal transition, as in wound healing, fibrosis and metastasis.	1.5E-22
MTORC1 SIGNALING	Genes up-regulated through activation of mTORC1 complex.	1.5E-22
APOPTOSIS	Genes mediating programmed cell death (apoptosis) by activation of caspases.	3.28E-19
OXIDATIVE PHOSPHORYLATION	Genes encoding proteins involved in oxidative phosphorylation.	3.77E-19

*by Gene Set Enrichment Analysis http://software.broadinstitute.org/gsea/msigdb/index.jsp , using experimentally validated miRNA targets, strong evidences, from http://mirtarbase.mbc.nctu.edu.tw.

Supplementary Table 3: Pathway enrichment using experimentally validated gene targets of miRNA cluster 3*

Gene Set Name	Pathway Description	FDR *Q* value
**Immunologic Signatures**		
GSE9601	Genes down-regulated in monocytes after HCMV infection: BAY 11-7082 versus Ly294002.	1.66E-14
GSE41176	Genes up-regulated in B lymphocytes: untreated versus anti-IgM for 1h.	8.11E-14
GSE16266	Genes up-regulated in mouse embryonic fibroblasts (MEF): control versus LPS.	3.4E-12
GSE22196	Genes down-regulated in skin gamma delta T cells: healthy versus obesity.	3.4E-12
GSE7219	Genes down-regulated in dendritic cell NIK NFkB2-KO versus dendritic cell NIK NFkB2-KO LPS and anti-CD40 stimulated.	5.45E-12
GSE14769	Genes down-regulated in comparison of unstimulated macrophage cells versus macrophage cells stimulated with LPS (TLR4 agonist) for 240 min.	8.32E-12
GSE24574	Genes down-regulated in BCL6 high follicular helper T cells versus naive T CD4 cells.	8.32E-12
GSE2585	Genes down-regulated in cortical thymic epithelial cells (cTEC) versus thymic macrophages.	8.32E-12
GSE27434	Genes down-regulated in T reg: wildtype versus DNMT1 knockout.	8.32E-12
GSE29618	Genes down-regulated in comparison of B cells versus monocytes.	8.32E-12
GSE29949	Genes up-regulated in brain: microglia versus dendritic cells.	8.32E-12
GSE3203	Genes up-regulated in lymph node B lymphocytes: untreated versus interferon beta.	8.32E-12
GSE42021	Genes up-regulated in T reg: peripheral lymph nodes versus thymic CD24 high.	8.32E-12
KAECH NAIVE VS DAY15 EFF CD8 TCELL	Genes down-regulated in comparison of naive versus memory CD8 T cells (day 40+ after LCMV-Armstrong infection).	8.32E-12
GSE6269	Genes down-regulated in comparison of peripheral blood mononuclear cells (PBMC) from patients with acute E. coli infection versus PBMC from patients with acute S. aureus infection.	1.96E-11
GSE14769	Genes down-regulated in comparison of unstimulated macrophage cells versus macrophage cells stimulated with LPS (TLR4 agonist) for 60 min.	4.54E-11
GSE17721	Genes up-regulated in comparison of dendritic cells (DC) stimulated with LPS (TLR4 agonist) at 12 h versus DC cells stimulated with CpG DNA (TLR9 agonist) at 12 h.	4.54E-11
GSE18203	Genes down-regulated in tumors established by injecting MC38 cells (colon cancer): control versus CpG oligodeoxynucleotide 1826.	4.54E-11
GSE19401	Genes up-regulated in ex vivo follicular dendritic cells from peripheral lymph nodes: naive versus immunized mice.	4.54E-11
GSE22935	Genes down-regulated in macrophages 48h after M. bovis BCG infection: wildtype versus MYD88 knockout.	4.54E-11
**Hallmark Gene Sets**		
TNFA SIGNALING VIA NFKB	Genes regulated by NF-kB in response to TNF.	1.27E-20
UV RESPONSE	Genes down-regulated in response to ultraviolet (UV) radiation.	2.04E-16
APOPTOSIS	Genes mediating programmed cell death (apoptosis) by activation of caspases.	2.04E-16
EPITHELIAL MESENCHYMAL TRANSITION	Genes defining epithelial-mesenchymal transition, as in wound healing, fibrosis and metastasis.	2.79E-14
G2M CHECKPOINT	Genes involved in the G2/M checkpoint, as in progression through the cell division cycle.	2.79E-14
P53 PATHWAY	Genes involved in p53 pathways and networks.	1.99E-13
MITOTIC SPINDLE	Genes important for mitotic spindle assembly.	1.22E-12
MTORC1 SIGNALING	Genes up-regulated through activation of mTORC1 complex.	1.22E-12
ESTROGEN RESPONSE EARLY	Genes defining early response to estrogen.	8.49E-12
PI3K AKT MTOR SIGNALING	Genes up-regulated by activation of the PI3K/AKT/mTOR pathway.	2.46E-11

*by Gene Set Enrichment Analysis http://software.broadinstitute.org/gsea/msigdb/index.jsp , using experimentally validated miRNA targets, strong evidences, from http://mirtarbase.mbc.nctu.edu.tw.

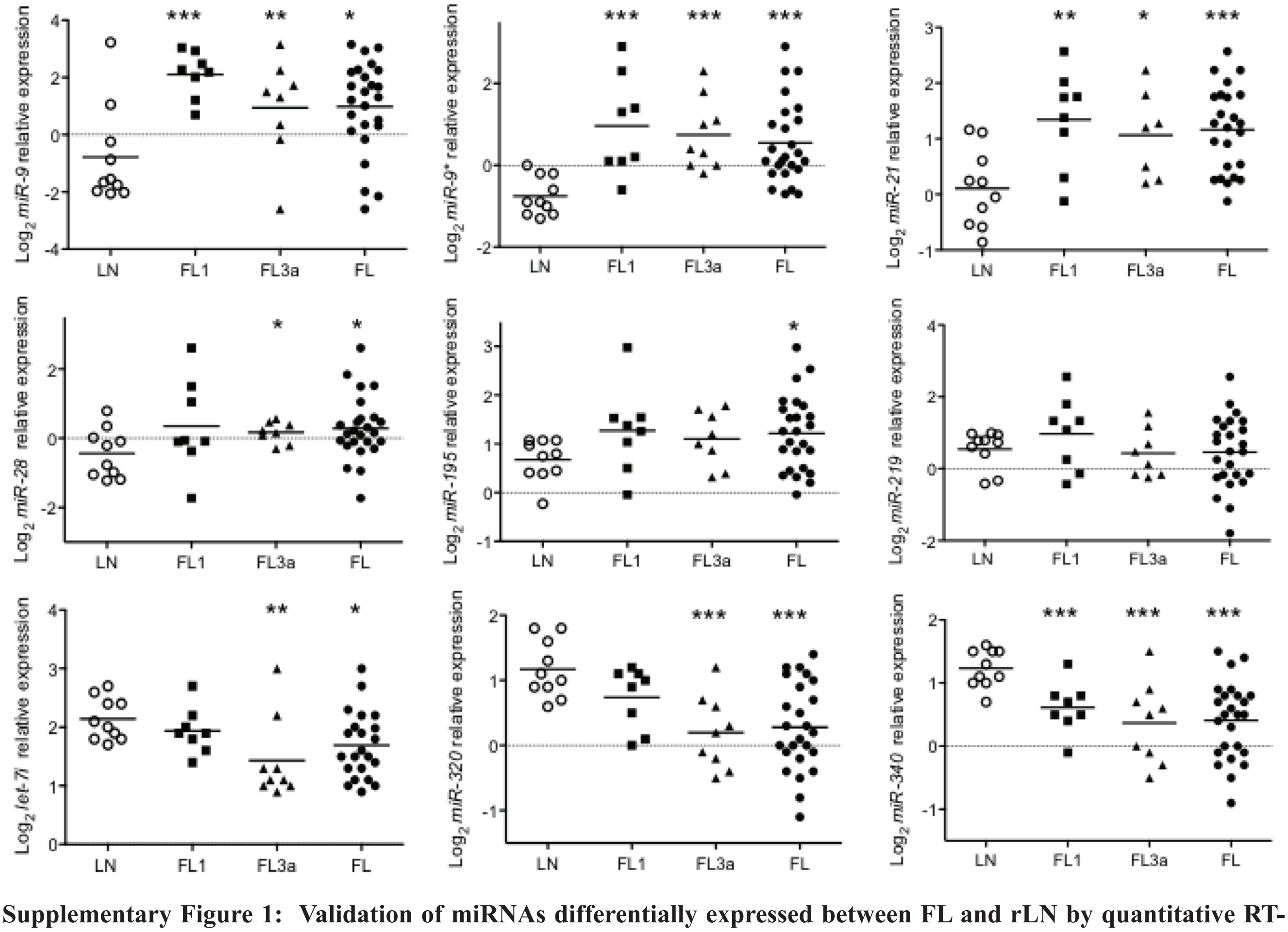

Supplementary Figure 1: Validation of miRNAs differentially expressed between FL and rLN by quantitative RT-PCR. Expression analysis of *miR-9*, *miR-9**, *miR-21*, *miR-28*, *miR-195*, *miR-219*, *let-7i*, *miR-320* and *miR-340* in ten reactive lymph nodes (rLN), eight follicular lymphoma (FL) grade 1, eight FL grade 3a and in altogether 25 FLs of grade 1, 2, 3a and 3b. The data are log_2 relative expression levels normalized with *RNU6B*. Statistical analysis was performed by *t*-test. Asterisks at the top of the graphs identify FL category significantly differentially expressed compared to rLNs: $^{*}P < 0.05$; $^{**}P < 0.01$; $^{***}P < 0.001$. R, relapsed; NR, not relapsed; n.a., not available.

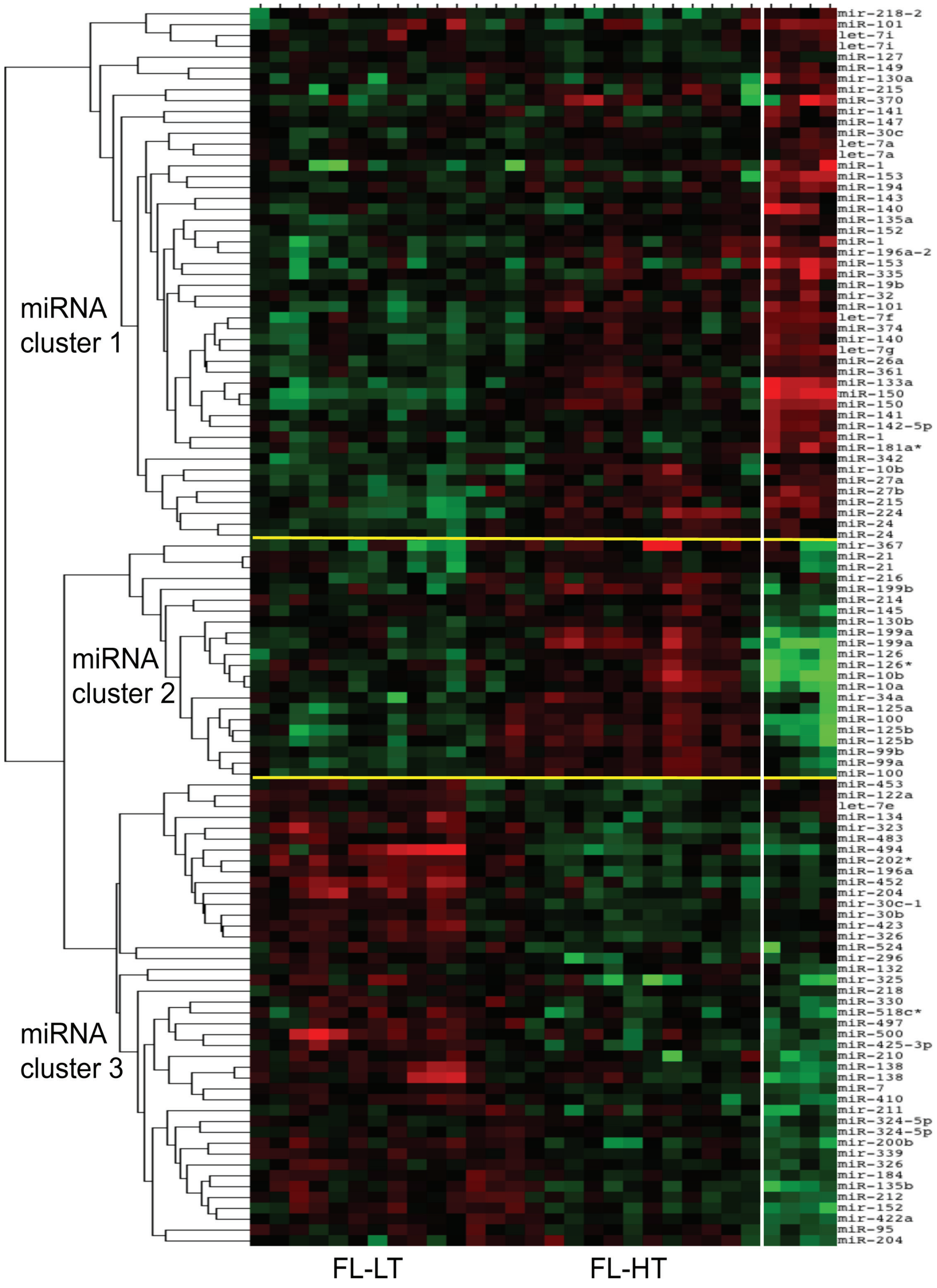

Supplementary Figure 2: MiRNAs expression profile in FLs, CD4⁺ T-cells and CD8⁺ T-cells samples; a copy of Figure 3 with all miRNAs differentially expressed. FL-HT, follicular lymphomas (FL) with high T-cell content. FL-LT, FLs with low T-cell content. The heat map describes the expression levels of miRNAs differentially expressed among three categories and 30 samples: 26 FLs, two $CD4^+$ and two $CD8^+$ T-cell samples (FDR 1%). FL-1, FL-2, FL-3a, FL-3b are FLs of grade 1, 2, 3a and 3b, respectively. Red, higher expression (log_2, +4); green, lower expression (log_2, –4).

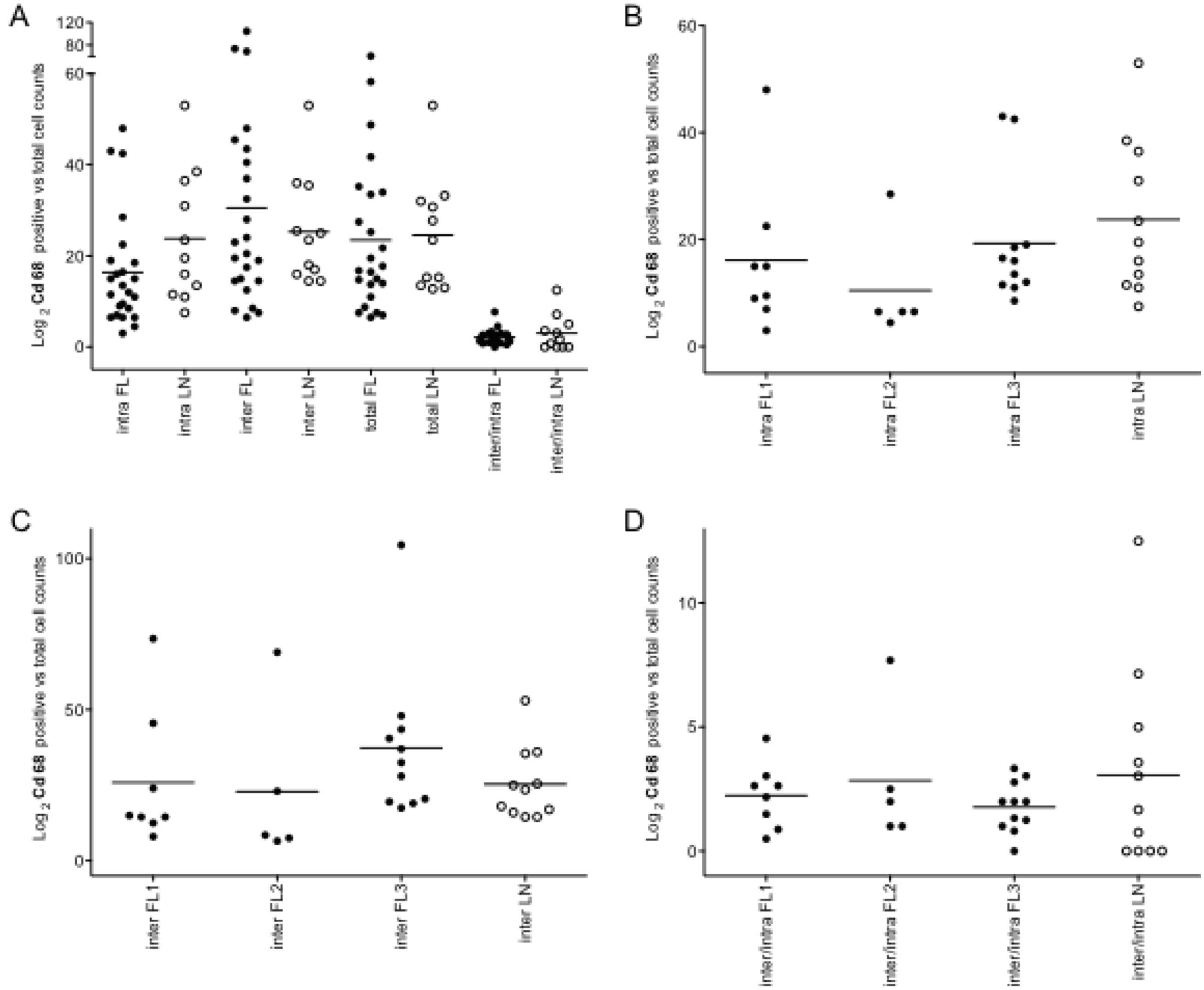

Supplementary Figure 3: Intrafollicular, interfollicular and total Cd68$^+$ cell counts in FLs and rLN by immunohistochemistry. Black circles, follicular lymphomas (FL); white circles, reactive lymph nodes (rLN). Intra, inter and total are intrafollicular, interfollicular and intrafollicular plus interfollicular Cd68$^+$ cell counts, respectively. FL1, FL2, FL3a, FL3b are FLs of grade 1, 2, 3a and 3b, respectively. Number of Cd68$^+$ cells in each FL and rLN sample was the average of cells counted in two consecutive slides. The average number of positive cells was normalized on the whole number of nuclei present in a slide. *P* values were calculated by *t*-test. (**A**) Cd68$^+$ cell counts in intrafollicular, interfollicular, total and interfollicular/intrafollicular ratio in FLs and rLNs. (**B**) Intrafollicular Cd68$^+$ cell counts in FL grades and rLN. (**C**) Interfollicular Cd68$^+$ cell counts in FL grades and LN. (**D**) Interfollicular/intrafollicular ratio of Cd68$^+$ cell counts in FL grades and rLN.

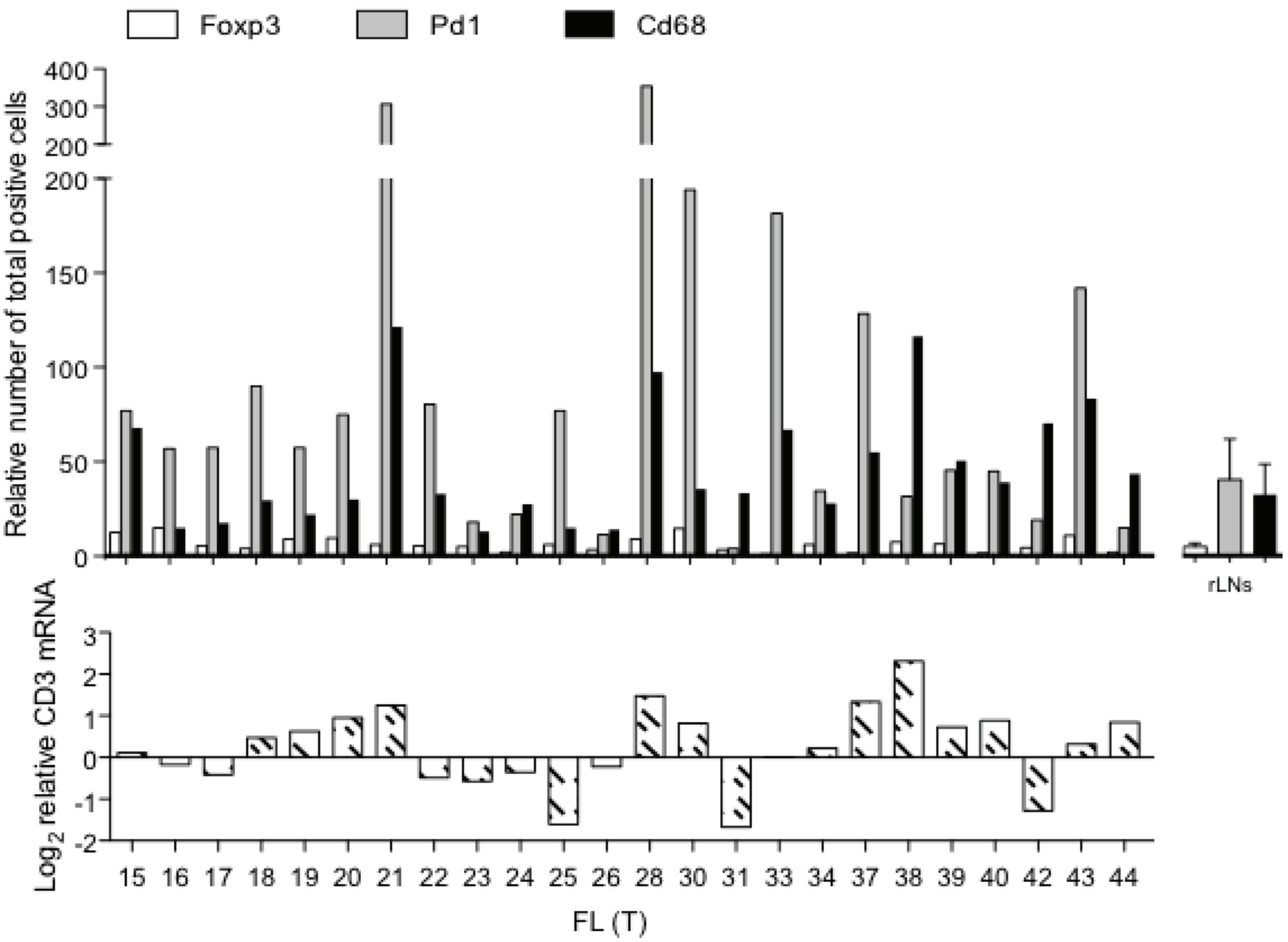

Supplementary Figure 4: Foxp3⁺, Pd1⁺ and Cd68+ total positive cells count in 24 FLs and 11 rLNs by immunohistochemistry. (**A**) Number of Foxp3⁺, PD1⁺ and CD68⁺ cells in each follicular lymphomas (FL) and reactive lymph nodes (rLN) samples was the average of cells counted in two consecutive slides. The average number of positive cells was normalized on the whole number of nuclei present in a slide. Bars and wiskers represent the average and the standard deviation of 11 rLNs cases. (**B**) Log_2 *CD3* mRNA level normalized to *GAPDH* in FLs by quantitative RT-PCR.

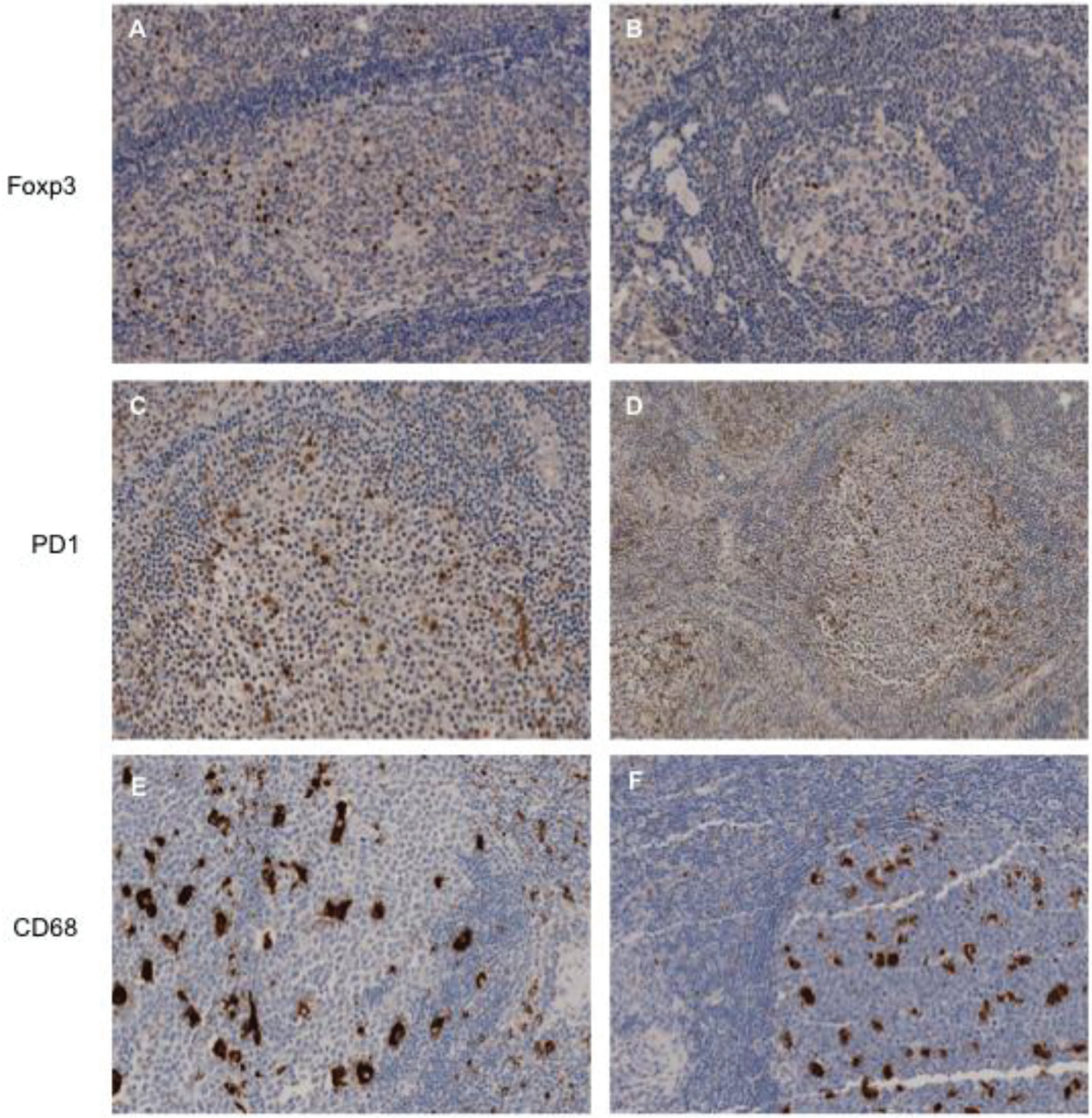

Supplementary Figure 5: Examples of Foxp3, PD1 and CD68 IHC staining in FL samples. (**A** and **B**) Prevalent intrafollicular distribution of Foxp3+ cells. (**C** and **D**), Prevalent intrafollicular distribution of PD1+ cells. (**E** and **F**) Intrafollicular distribution of CD68+ cells.

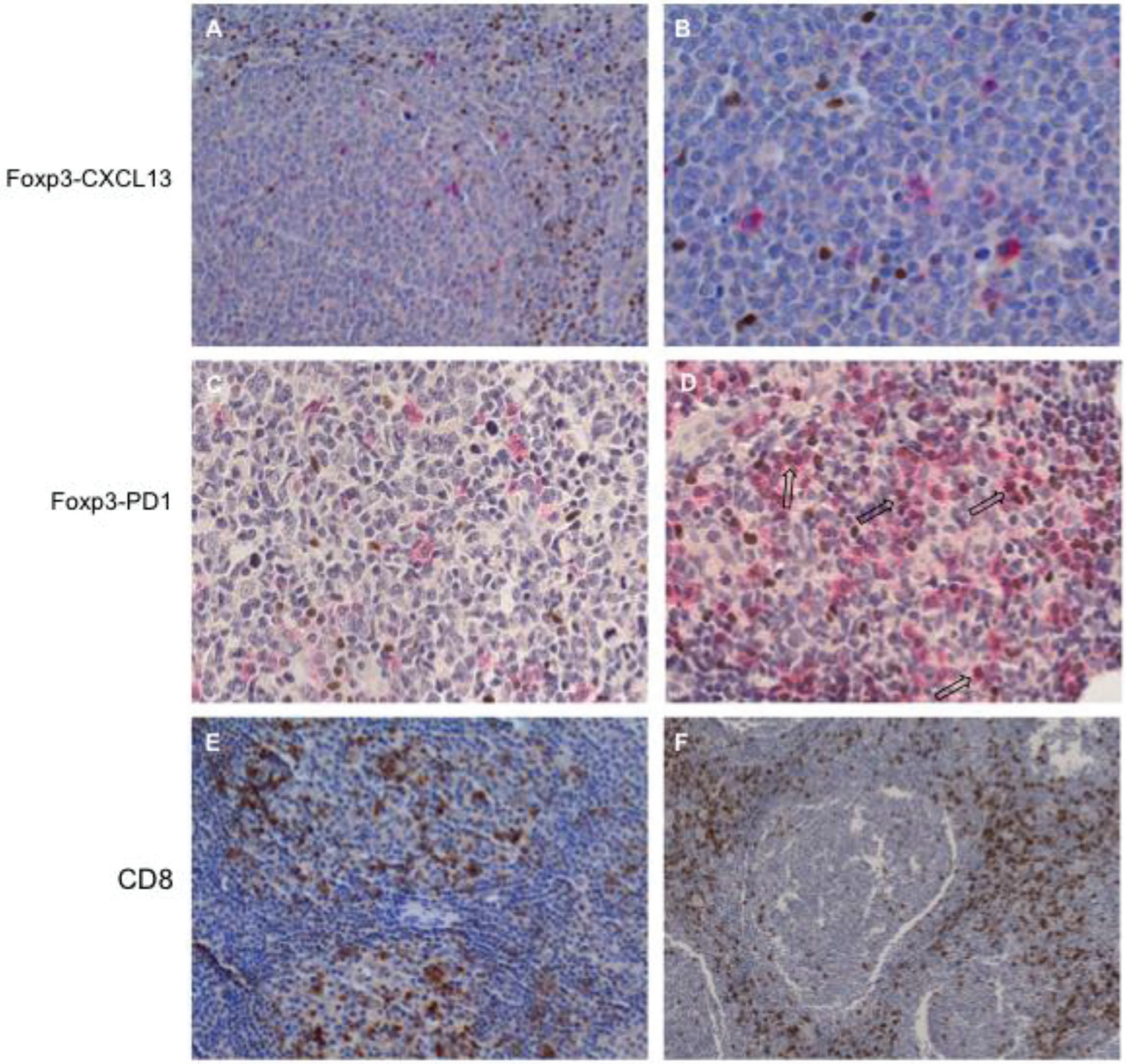

Supplementary Figure 6: Examples of Foxp3-CXCL13 and Foxp3-PD1 and of CD8 IHC staining in FL samples. For double staining, Foxp3 (nuclear) was revealed by horsereadish peroxidase and second antibody by alkaline phosphatase (cytoplasmic). (**A** and **B**) Independent expression of Foxp3 and CXCL13 in two FL samples. (**C**) Independent expression of Foxp3 and PD1 in one FL sample. (**D**) Foxp3 and PD1 co-expression in some GC cells of FL case T20, as indicated by the arrows. (**E**) Intrafollicular invasion by $CD8^+$ cells. (**F**) Interfollicular distribution of $CD8^+$ cells.

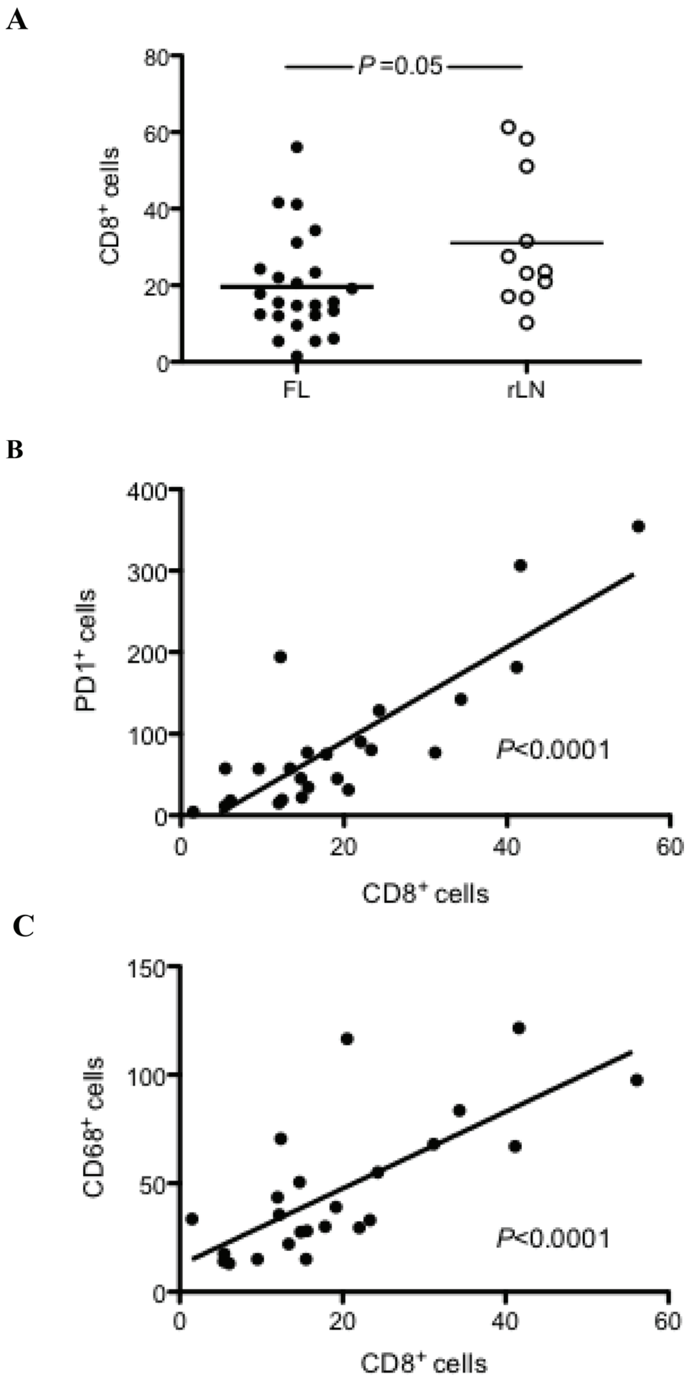

Supplementary Figure 7: CD8$^+$ cells number is lower in FL than in rLN and correlates with PD1$^+$ and CD68$^+$ cells counts in FLs. Black circle, follicular lymphoma, FL; empty circle, reactive lymph nodes, rLN. (**A**) CD8$^+$ cells number in FL and rLN. Horizontal straight line represents the average value. (**B**) Direct correlation between CD8$^+$ and PD1$^+$ cells number in FL. Straight line and *P* value indicate a significant correlation between CD8$^+$ and PD1$^+$ cell number in FLs. (**C**) Direct correlation between CD8$^+$ and CD68$^+$ cell number in FLs. Straight line and *P* value indicate a significant correlation between CD8$^+$ and CD68$^+$ cell number in FLs.

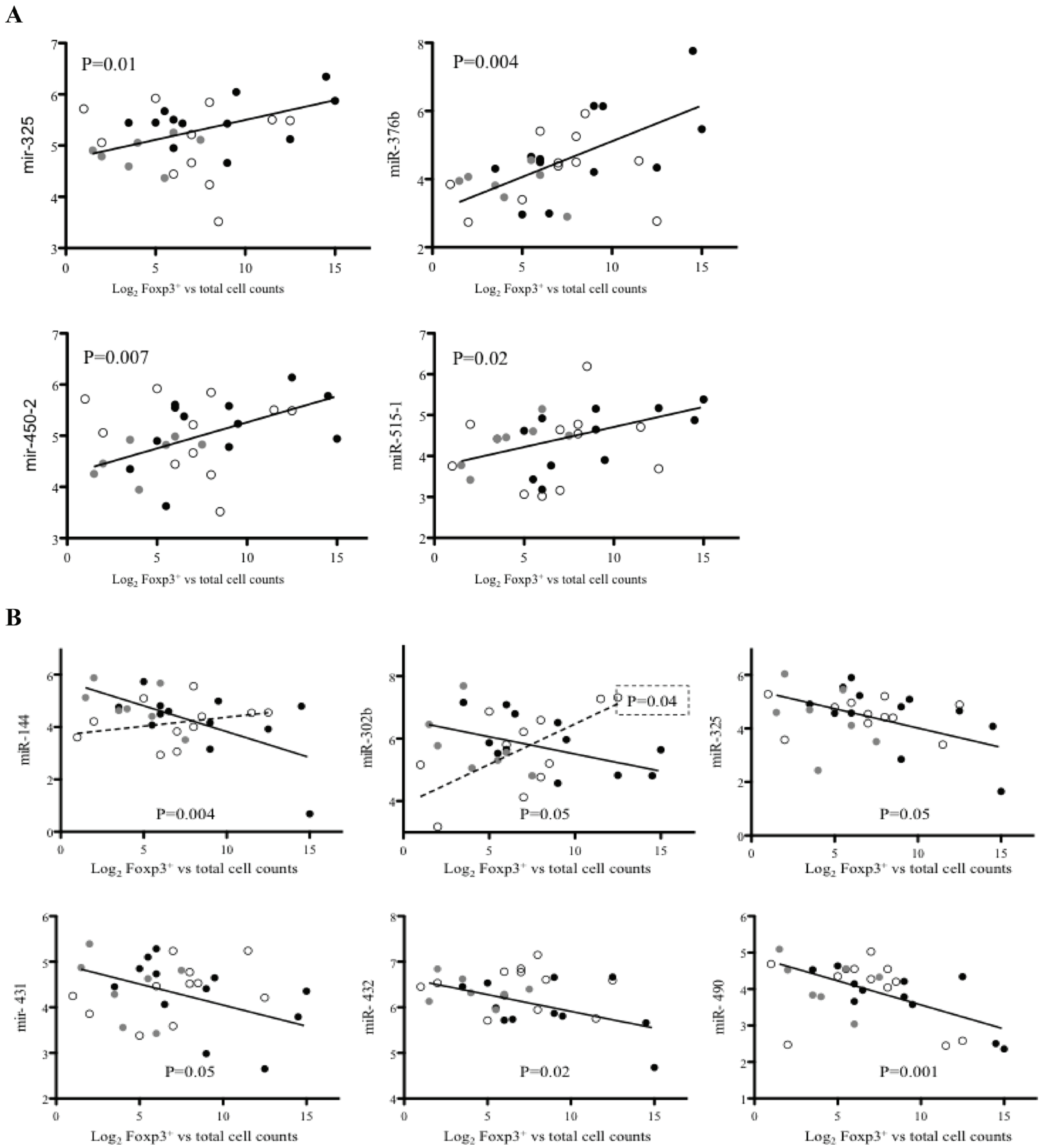

Supplementary Figure 8: Correlation between ten miRNAs and total Foxp3^{+} cell counts in FLs and rLNs. Black circle, not relapsed follicular lymphomas (FL); grey circle, relapsed FLs; empty circle, reactive lymph nodes (rLN). (**A**) Direct correlation between *mir-325*, *miR-376b*, *mir-450-2*, *mir-515-1* and Foxp3^{+} cell counts. (**B**) Inverse correlation between *miR-144*, *miR-302b*, *miR-325*, *mir-431*, *miR-432*, *miR-490* and Foxp3^{+} cells count. Straight line and P value indicate a significant correlation between miRNA level and Foxp3^{+} cell counts in FLs. Dashed line indicates a significant correlation between miRNA level and Foxp3^{+} cell counts in rLNs.
